# Effect of Bevacizumab in Combination With Standard Oxaliplatin-Based Regimens in Patients With Metastatic Colorectal Cancer: A Randomized Clinical Trial

**DOI:** 10.1001/jamanetworkopen.2021.18475

**Published:** 2021-07-26

**Authors:** Antonio Avallone, Maria C. Piccirillo, Guglielmo Nasti, Gerardo Rosati, Chiara Carlomagno, Elena Di Gennaro, Carmela Romano, Fabiana Tatangelo, Vincenza Granata, Antonino Cassata, Lucrezia Silvestro, Alfonso De Stefano, Luigi Aloj, Valeria Vicario, Anna Nappi, Alessandra Leone, Domenico Bilancia, Laura Arenare, Antonella Petrillo, Secondo Lastoria, Ciro Gallo, Gerardo Botti, Paolo Delrio, Francesco Izzo, Franco Perrone, Alfredo Budillon

**Affiliations:** 1 Experimental Clinical Abdominal Oncology Unit, Istituto Nazionale Tumori–Istituto di Ricovero e Cura a Carattere Scientifico (IRCCS), Fondazione G. Pascale, Napoli, Italy; 2 Clinical Trials Unit, Istituto Nazionale Tumori–IRCCS, Fondazione G. Pascale, Napoli, Italy; 3 Innovative Therapy for Abdominal Metastases, IRCCS, Fondazione G. Pascale, Napoli, Italy; 4 Medical Oncology Unit, S. Carlo Hospital, Potenza, Italy; 5 Department of Clinical Medicine and Surgery, University Federico II, Naples, Italy; 6 Experimental Pharmacology Unit, Istituto Nazionale Tumori–IRCCS, Fondazione G. Pascale, Napoli, Italy; 7 Pathology Unit, Istituto Nazionale Tumori–IRCCS, Fondazione G. Pascale, Napoli, Italy; 8 Radiology Unit, Istituto Nazionale Tumori–IRCCS, Fondazione G. Pascale, Napoli, Italy; 9 Nuclear Medicine Unit, Istituto Nazionale Tumori–IRCCS, Fondazione G. Pascale, Napoli, Italy; 10 currently affiliated with Department of Radiology, University of Cambridge School of Clinical Medicine, Cambridge, United Kingdom; 11 Università della Campania Luigi Vanvitelli, Napoli, Italy; 12 Colorectal Oncological Surgery, Istituto Nazionale Tumori–IRCCS, Fondazione G. Pascale, Napoli, Italy; 13 Hepatobiliary Surgery Unit, Istituto Nazionale Tumori–IRCCS, Fondazione G. Pascale, Napoli, Italy

## Abstract

**Importance:**

Although bevacizumab is a standard of care in combination treatments for metastatic colorectal cancer (mCRC), its clinical benefit has been limited.

**Objective:**

To determine whether sequential scheduling of bevacizumab administration in combination with chemotherapy improves treatment efficacy in patients with mCRC, in keeping with the tumor vascular normalization hypothesis.

**Design, Setting, and Participants:**

This open-label, randomized clinical phase 3 trial was conducted from May 8, 2012, to December 9, 2015, at 3 Italian centers. Patients aged 18 to 75 years with unresectable, previously untreated, or single line–treated mCRC were recruited. Follow-up was completed December 31, 2019, and data were analyzed from February 26 to July 24, 2020.

**Interventions:**

Patients received 12 biweekly cycles of standard oxaliplatin-based regimens (modified FOLFOX-6 [levo–folinic acid, fluorouracil, and oxaliplatin]/modified CAPOX [capecitabine and oxaliplatin]) plus bevacizumab administered either on the same day as chemotherapy (standard arm) or 4 days before chemotherapy (experimental arm).

**Main Outcomes and Measures:**

The primary end point was the objective response rate (ORR) measured with Response Evaluation Criteria in Solid Tumors, version 1.1. Secondary end points included progression-free survival, overall survival, safety, and quality of life (QOL).

**Results:**

Overall, 230 patients (136 men [59.1%]; median age, 62.3 [interquartile range, 53.3-67.6] years) were randomly assigned to the standard arm (n = 115) or the experimental arm (n = 115). The median duration of follow-up was 68.3 (95% CI, 61.0-70.0) months. No difference in ORR (57.4% [95% CI, 47.8%-66.6%] in the standard arm and 56.5% [95% CI, 47.0-65.7] in the experimental arm; *P* = .89) or progression-free survival (10.5 [95% CI, 9.1-12.3] months in the standard arm and 11.7 [95% CI, 9.9-12.9] months in the experimental arm; *P* = .15) was observed. However, the median overall survival was 29.8 (95% CI, 22.5-41.1) months in the experimental arm compared with 24.1 (95% CI, 18.6-29.8) months in the standard arm (adjusted hazard ratio, 0.73; 95% CI, 0.54-0.99; *P* = .04). Moreover, the experimental arm was associated with a significant reduction in the rate of severe diarrhea (6 [5.3%] vs 19 [16.5%]; *P* = .006) and nausea (2 [1.8%] vs 8 [7.0%]; *P* = .05) and improved physical functioning (mean [SD] change from baseline, 0.65 [1.96] vs −7.41 [2.95] at 24 weeks; *P* = .02), and constipation scores (mean [SD] change from baseline, −17.2 [3.73] vs −0.62 [4.44]; *P* = .003).

**Conclusions and Relevance:**

In this randomized clinical trial, sequential administration of bevacizumab plus chemotherapy did not improve ORR, the primary end point. However, the overall survival advantage, fewer adverse effects, and better health-related QOL associated with sequential bevacizumab administration might provide the basis for exploring antiangiogenic combination treatments with innovative perspectives.

**Trial Registration:**

EudraCT Identifier: 2011-004997-27; ClinicalTrials.gov Identifier: NCT01718873

## Key Points

**Question:**

Does sequential scheduling of bevacizumab administration in combination with chemotherapy improve treatment efficacy in patients with metastatic colorectal cancer in keeping with the tumor vascular normalization hypothesis?

**Findings:**

In this phase 3 randomized clinical trial of 230 patients, the primary end point objective response rate did not significantly differ between the sequential and the concomitant schedule of bevacizumab administration in combination with standard oxaliplatin-based regimens. However, the sequential schedule of bevacizumab administration was associated with longer overall survival, fewer adverse effects, and better health-related quality of life.

**Meaning:**

Sequential bevacizumab scheduling plus chemotherapy might be relevant to optimize therapeutic efficacy and to explore antiangiogenic combination treatments with innovative perspectives.

## Teaser

This randomized clinical trial compares outcomes of concomitant and sequential administration of bevacizumab in combination with standard oxaliplatin-based regimens in untreated or single line–treated patients with metastatic colorectal cancer.

## Introduction

The treatment of metastatic colorectal cancer (mCRC) has improved significantly with the addition of new cytotoxic drugs and biological agents to fluoropyrimidine regimens.^1^ However, mCRC remains incurable in most cases, and no novel drugs have been included in the therapeutic arsenal against this disease in recent years. In this scenario, optimization of the combined use of available drugs with proven efficacy provides a relevant challenge.

Angiogenesis has emerged as a crucial hallmark of cancer development, becoming a key target for cancer treatment of various solid tumors, including CRC.^2,3^ Tumor angiogenesis is highly dependent on the activity of vascular endothelial growth factor (VEGF) and is characterized by structural and functional vasculature abnormalities leading to a hostile microenvironment that impedes drug delivery and fuels tumor invasion and treatment resistance.^4,5,6^ Bevacizumab, a humanized monoclonal antibody directed against VEGF, is the standard of care for the treatment of mCRC in the first- and second-line setting in combination with conventional chemotherapy.^7^

The clinical benefit of bevacizumab combination treatments, however, has proven to be rather limited and occasionally questioned in mCRC.^8,9,10^ Moreover, the disappointing clinical results of bevacizumab monotherapy indicate that its mechanism of action is not confined to tumor starvation as originally postulated.^11,12,13^ Indeed, the mechanism by which bevacizumab improves the efficacy of chemotherapy remains under active investigation.^14^

Several preclinical and clinical studies suggest that an important therapeutic mechanism of anti-VEGF agents is the induction of tumor vascular normalization.^15^ Tumor vascular normalization occurs 4 to 5 days after bevacizumab treatment. Sequential scheduling aimed at normalizing vasculature before delivery of chemotherapeutics could be of considerable importance to optimize the efficacy of combination treatment.^5,16,17^

Avallone et al^18^ demonstrated the critical role of bevacizumab scheduling in patients with locally advanced rectal cancer (BRANCH [Bevacizumab, Radiotherapy and Chemotherapy] trial). Bevacizumab administered 4 days before presurgical chemoradiotherapy resulted in a higher rate of complete tumor regression and a better toxicity profile compared with the traditional concomitant schedule. Following up on these findings, an academic multicenter randomized phase 3 study was designed to compare concomitant and sequential scheduling of bevacizumab in combination with standard oxaliplatin-based regimens in untreated or single line–treated patients with mCRC.^19^

## Methods

### Study Design and Patient Selection

The OBELICS (Optimization of Bevacizumab Scheduling Within Chemotherapy) study was an open-label, 2-arm, randomized clinical phase 3 trial conducted in 3 Italian centers. The trial was approved by the ethics committees of the participant sites, and all patients provided written informed consent. This study followed the Consolidated Standards of Reporting Trials (CONSORT) reporting guideline.

Randomization was performed by a centrally computer-generated minimization procedure accounting for center, Eastern Cooperative Oncology Group (ECOG) performance status (0 vs 1), previous chemotherapy for advanced disease (yes vs no), and number of metastatic sites (1 vs >1) to balance the distribution of these factors between the 2 treatment arms. Patients aged 18 to 75 years with histologically confirmed unresectable mCRC regardless of *RAS* (OMIM 190070 and 164790) mutational status, with no more than 1 previous treatment line, and with an ECOG performance status of 1 or less were eligible. Adequate hematologic, hepatic, and renal function and adequate recovery from recent surgery (at least 28 days after a major surgery or biopsy) were required. The study protocol (Supplement 1) has been reported previously.^19^ Mutational status of tumor *RAS*, *KRAS* (OMIM 190070) and *NRAS* (OMIM 164790) exons 2 to 4, as well as *BRAF* codon 600 (OMIM 164757), were assessed on DNA extracted from archival tissue specimens from the primary tumor or metastases as reported elsewhere.^20^

### Treatment Plan

Patients were randomized (1:1) to receive 12 biweekly cycles of a modified FOLFOX-6 regimen (intravenous oxaliplatin, 85 mg/m^2^, on day 1, followed by intravenous levo–folinic acid, 200 mg/m^2^, plus bolus fluorouracil, 400 mg/m^2^, and a 46-hour intravenous administration of fluorouracil, 2400 mg/m^2^) or a modified CAPOX regimen (intravenous oxaliplatin, 85 mg/m^2^, on day 1 plus oral capecitabine, 1000 mg/m^2^, twice daily on days 1 to 10) every 2 weeks^21^ for 12 cycles. Bevacizumab (5 mg/kg) was administered on the same day as chemotherapy (standard arm) or 4 days before (experimental arm).

Maintenance treatment with bevacizumab with or without fluoropyrimidines was planned in both arms until progression or unacceptable toxic effects. In case of prespecified adverse events, treatment modifications were allowed as outlined in the study protocol (Supplement 1).^19^ Surgery could be performed if a patient became eligible for curative resection of metastatic disease; the choice of treatment after surgery was at the discretion of the investigator.

### Clinical Outcome

Objective response rate (ORR) according to Response Evaluation Criteria in Solid Tumors (RECIST), version 1.1, was the primary end point and was defined as the number of complete plus partial responses divided by the number of enrolled patients. Objective response was assessed by computed tomographic scan or other appropriate imaging at weeks 12 and 24 from randomization and every 3 months thereafter. Disease control rate was calculated by adding complete and partial responses and stable disease. Secondary end points included progression-free survival (PFS), overall survival (OS), toxic effects, and quality of life (QOL). Progression-free survival was defined as the time from randomization to the date of progression or death, whichever occurred first. Patients without progression were censored on the date of the last follow-up visit. Overall survival was defined as the time from randomization to the date of death. Patients alive at the time of the final analysis were censored on the date of the last follow-up information available. Toxic effect were scored according to the National Cancer Institute Common Toxicity Criteria for Adverse Events (CTCAE), version 4.0. Quality of life was assessed by the European Organization for Research and Treatment of Cancer Quality of Life Questionnaire–Core30, version 3.0, questionnaire at baseline and at week 12 and 24 during treatment, in both arms.

Biomarker studies on tumor and blood samples (Supplement 1) and [18^F^]-2-fluoro-2-deoxy-d-glucose positron emission tomography evaluation were also planned, as reported previously.^19^ The results of correlative studies will be described separately.

### Statistical Analysis

Data were analyzed from February 26 to July 24, 2020. Study sample size was calculated considering different possible proportions of patients enrolled for first- or second-line treatment. Assuming an ORR of 40% in the first line and 20% in the second line, an anticipated odds ratio of 2.25, 80% power, and 5% 2-sided α error, and considering that the needed sample size increases with the lowering of the ORR expected in the standard arm, a sample size of 230 patients was determined to be adequate to verify the study hypothesis also in the extreme case of 75% of patients enrolled for second-line chemotherapy (that corresponds to an expected ORR of 25% with standard treatment).

All analyses were performed based on an intention-to-treat strategy. The ORRs in the 2 arms were reported with 95% CIs and were compared with the χ^2^ test in a 2 × 2 contingency table (responders and nonresponders × treatment arms). The median duration of disease control was estimated using time-to-event analysis with a Cox proportional hazards regression model, with an event considered as progression after a complete or partial response or stable disease. Median follow-up was calculated according to the reverse Kaplan-Meier technique of Schemper.^45^ Survival curves were described according to the Kaplan-Meier product-limit method. Estimated hazard ratios (HRs) and their 95% CIs were calculated using the Cox proportional hazard model adjusted by ECOG performance status, previous chemotherapy for advanced disease, number of metastatic sites, and *RAS* mutational status as covariates and stratified by type of chemotherapy regimen chosen by the investigator. First-order interactions between treatment and main prognostic variables were tested by the likelihood ratio test of 2 nested models with and without interaction. Patients who received at least 1 study drug dose were eligible for safety analysis. For each patient and type of toxic effects, the worst degree observed during treatment was used for analysis. Differences between study arms in the whole pattern of toxic effects (all grades) for each item were analyzed with a linear permutation test accounting for ordinal nature of data (linear rank test). Occurrence of any grade (>0) and severe (>2) toxic effects were also compared between the 2 arms with the χ^2^ test. Mean changes from baseline in global health status and QOL items at each point were compared between the 2 arms in a linear regression model, adjusted by the previous covariates and the baseline QOL scores.^22^ The best QOL response from baseline for each domain or symptom was calculated by defining a score change of at least 10 points from baseline as clinically relevant and comparing the 2 arms with the χ^2^ test.^23^ No adjustment was applied for multiple comparisons, owing to the explorative nature of secondary and subgroup analyses. Follow-up was completed on December 31, 2019. Statistical analyses were performed using STATA MP, version 14.1 (StataCorp LLC); 2-sided *P* < .05 indicated statistical significance.

## Results

**Figure 1.**
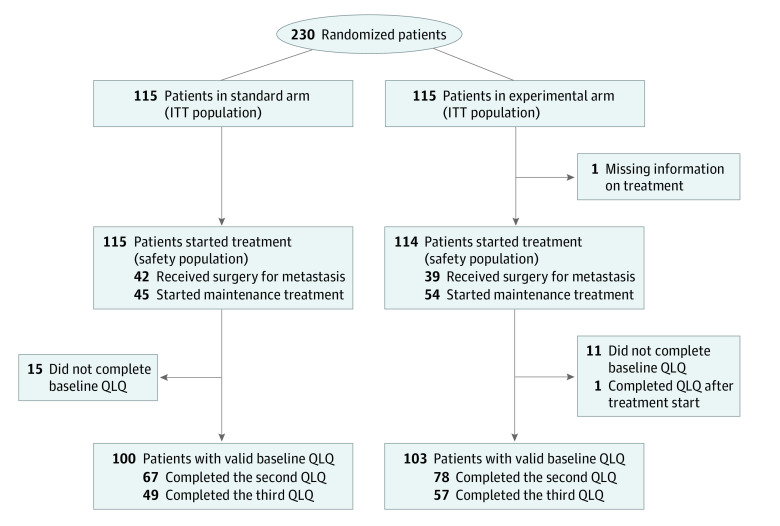
CONSORT Diagram Showing the Trial Progress ITT indicates intention to treat; QLQ, Quality of Life Questionnaire–Core30.

From May 8, 2012, to December 9, 2015, 230 patients (136 men [59.1%] and 94 women [40.9%]; median age, 62.3 [interquartile range (IQR), 53.3-67.6] years) were randomized to the standard arm (n = 115) or to the experimental arm (n = 115). Information on treatment was missing for 1 patient in the experimental arm who was not included in the safety analysis (Figure 1). Baseline patient characteristics were well balanced between the treatment arms (Table 1), but a higher proportion of patients with *RAS* mutations were included in the experimental arm (71 [61.7%] vs 54 [47.0%]; *P* = .06).

**Table 1.** Baseline Patient Characteristics

Characteristic	Treatment group (N = 230)^a^	
	Standard (n = 115)	Experimental (n = 115)
Age, median (IQR), y	63 (56-68)	61 (53-68)
Sex		
Men	67 (58.3)	69 (60.0)
Women	48 (41.7)	46 (40.0)
ECOG performance status		
0	91 (79.1)	90 (78.3)
1	24 (20.9)	25 (21.7)
Previous treatments for advanced disease		
0	107 (93.0)	108 (93.9)
1	8 (7.0)	7 (6.1)
Metastatic sites		
1	61 (53.0)	60 (52.2)
>1	54 (47.0)	55 (47.8)
Time to metastasis		
Synchronous	92 (80.0)	93 (80.9)
Metachronous	23 (20.0)	22 (19.1)
*RAS* status		
Wild-type	54 (47.0)	37 (32.2)
Altered	54 (47.0)	71 (61.7)
Unknown	7 (6.1)	7 (6.1)
*BRAF* status		
Wild-type	36 (31.3)	25 (21.7)
Altered	3 (2.6)	2 (1.7)
Unknown	76 (66.1)	88 (76.5)
Primary site		
Right colon	32 (27.8)	30 (26.1)
Left colon	46 (40.0)	49 (42.6)
Rectum	36 (31.3)	35 (30.4)
Unknown	1 (0.9)	1 (0.9)
Chemotherapy regimen		
Modified FOLFOX-6	88 (76.5)	79 (68.7)
Modified CAPOX	27 (23.5)	36 (31.3)

Abbreviations: CAPOX, capecitabine and oxaliplatin; ECOG, Eastern Cooperative Oncology Group; FOLFOX-6, levo–folinic acid, fluorouracil, and oxaliplatin; IQR, interquartile range.

^a^ Unless indicated otherwise, data are expressed as No. (%) of patients. Percentages have been rounded and may not total 100.

Most of the patients (167 [72.6%]) received the modified FOLFOX-6 regimen. The median number of cycles was 12 (IQR, 10-12) in both arms. More patients in the standard arm required treatment delay (30 [26.1%] vs 22 [19.3%]) and dose reduction (14 [12.2%] vs 6 [5.3%]), whereas more patients completed treatment induction in the experimental arm (83 [72.8%] vs 78 [67.8%]) (eTable 1 in Supplement 2). Forty-four patients in the standard arm (38.3%) and 52 patients in the experimental arm (45.2%) received maintenance therapy, with a median number of cycles administered per patient of 4 (IQR, 2-6) and 6 (IQR, 4-8), respectively, and with most of these patients receiving bevacizumab plus fluoropyrimidines in both arms (38 of 44 [86.4%] and 46 of 52 [88.5%], respectively) (eTable 1 in Supplement 2).

**Figure 2.**
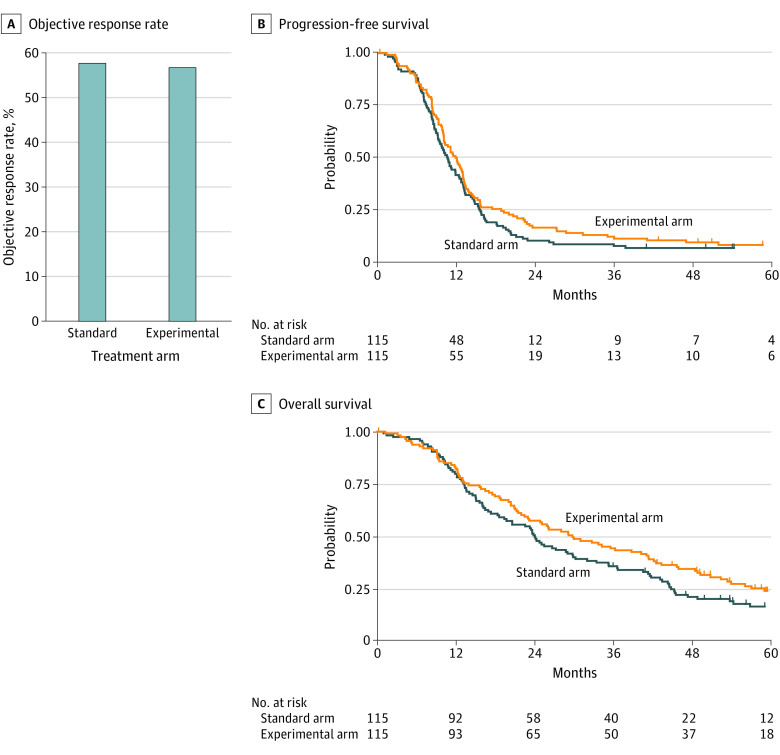
Objective Response Rate and Survival in Intention-to-Treat Population A, Objective response rate (ORR) measured with Response Evaluation Criteria in Solid Tumors, version 1.1, was 57.4% (95% CI, 47.8%-66.6%) in the standard arm and 56.5% (95% CI, 47.0%-65.7%) in the experimental arm. Odds ratio of response of experimental vs standard treatment was 0.96 (95% CI, 0.55-1.68; *P* = .89). B, Data are shown in the intention-to-treat population at the first data cutoff (December 31, 2019). Progression-free survival was 10.5 (95% CI, 9.1-12.3) months in the standard arm vs 11.7 (95% CI, 9.9-12.9) months in the experimental arm. The adjusted hazard ratio (HR) for the experimental arm vs the standard arm was 0.81 (95% CI, 0.62-1.08; *P* = .15). C, Overall survival at the median follow-up of 68.3 [interquartile range, 57.5-70.9] months was 24.1 (95% CI, 18.6-29.8) months in the standard arm vs 29.8 (95% CI, 22.5-41.1) months in the experimental arm (HR, 0.73; 95% CI, 0.54-0.99; *P* = .04).

No difference in ORR was observed between the 2 arms with an odds ratio of response for experimental vs standard treatment equal to 0.96 (95% CI, 0.55-1.68; *P* = .89) (Figure 2A). Complete or partial responses were reported in 66 patients in the standard arm (57.4%; 95% CI, 47.8%-66.6%) and 65 patients in the experimental arm (56.5%; 95% CI, 47.0%-65.7%) (Table 2). The disease control rate (103 [89.6%] for the standard arm vs 107 [93.0%] for the experimental arm) (Table 2) and median duration of disease control (6.6 [IQR, 5.3-8.6] months for the standard arm vs 7.1 [IQR, 5.7-9.0] months for the experimental arm) were also similar. Overall, a better ORR was observed in the *RAS* wild-type subgroups (35 of 54 [64.8%] for the standard arm and 26 of 37 [70.3%] for the experimental arm), with no difference between the 2 arms according to *RAS* status (29 of 54 [53.7%] vs 37 of 71 [52.1%] for *RAS* variant subgroups and 1 of 7 [14.3%] vs 2 of 7 [28.6%] for *RAS* unknown subgroups in the standard and experimental arms, respectively) (eTable 2 in Supplement 2). The number of *BRAF*-altered tumors was too small to allow correlation with outcomes. The timing of the evaluation of the objective response was similar between the 2 arms (eFigure 1 in Supplement 2).

**Table 2.** Outcomes Summary

Outcome	Treatment group		OR or HR (95% CI)	*P* value
	Standard (n = 115)	Experimental (n = 115)		
**Primary outcome**				
Objective response rate				
Response, No. (%) [95% CI]	66 (57.4) [47.8-66.6]	65 (56.5) [47.0-65.7]	OR, 0.96 (0.55-1.68)	.89
Complete response, No. (%)	3 (2.6)	3 (2.6)	NA	NA
Partial response, No. (%)	63 (54.8)	62 (53.9)	NA	
Stable disease, No. (%)	37 (32.2)	42 (36.5)	NA	
Progressive disease, No. (%)	7 (6.1)	3 (2.6)	NA	
Not evaluable, No. (%)	5 (4.3)	5 (4.3)	NA	
**Secondary outcomes**				
Disease control rate, No. (%) [95% CI]	103 (89.6) [82.5-94.5]	107 (93.0) [86.8-96.9]	OR, 1.56 (0.56-4.58)	.35
PFS, median (95% CI), mo	10.5 (9.1-12.3)	11.7 (9.9-12.9)	HR, 0.81 (0.62-1.08)	.15
OS, median (95% CI), mo	24.1 (18.6-29.8)	29.8 (22.5-41.1)	HR, 0.73 (0.54-0.99)	.04
Global QOL status mean change from baseline				
12-week	1.94	8.92	NA	.05
24-week	0.15	6.32	NA	.36
Global QOL status best response, No./total No. (%)				.41
Improved	33/77 (42.9)	40/86 (46.5)	NA	NA
Stable	20/77 (26.0)	27/86 (31.4)	NA	
Worse	24/77 (31.2)	19/86 (22.1)	NA	
Toxic effects				
Any grade	113 (98.3)	108 (94.7)	OR, 0.38 (0.36-2.40)	.24
Severe	75 (65.2)	65 (57.0)	OR, 0.72 (0.41-1.28)	.23

Abbreviations: HR, hazard ratio; NA, not applicable; OR, odds ratio; OS, overall survival; PFS, progression-free survival; QOL, quality of life.

No significant differences in the proportion of patients who underwent resection of metastases and in radical resection rate were observed between the 2 arms (eTable 3 in Supplement 2). At the end of follow-up (December 31, 2019), with a median follow-up of 68.3 (95% CI, 61.0-70.0) months, 211 PFS events occurred, 107 in the standard arm and 104 in the experimental arm. The median PFS was 10.5 (95% CI, 9.1-12.3) and 11.7 (95% CI, 9.9-12.9) months for the standard and experimental arms, respectively, with an adjusted HR of 0.81 (95% CI, 0.62-1.08; *P* = .15) (Figure 2B). The treatment effect on PFS was consistent across all clinical and molecular subgroups (eFigure 2 in Supplement 2). Overall, 182 patients died, 95 in the standard arm and 87 in the experimental arm. The median OS was 24.1 (95% CI, 18.6-29.8) and 29.8 (95% CI, 22.5-41.1) months for the standard and experimental arms, respectively, with an adjusted HR of 0.73 (95% CI, 0.54-0.99; *P* = .04) (Figure 2C). Overall survival benefit in the experimental arm was homogeneous in all clinical and molecular subgroups (eFigure 3 in Supplement 2).

The number of patients undergoing subsequent systemic therapies was similar in both arms (eTable 4 in Supplement 2). Among patients receiving second-line treatment, a significantly higher proportion of patients received a regimen containing anti–epidermal growth factor receptor (EGFR) in the standard arm (31 [27.0%] vs 21 [18.3%]) and a significantly higher proportion received an anti-VEGF–containing regimen in the experimental arm (49 [42.6%] vs 31 [27.0%]) as a consequence of the imbalance of patients in the *RAS* wild-type subgroups in the 2 arms. No significant difference in the distribution of subsequent treatment lines was observed between the 2 arms.

No unexpected toxic effects were reported. Adverse events occurring in at least 3% of patients are reported in Table 3 (complete information is provided in eTable 5 in Supplement 2). Patients receiving standard treatment experienced significantly more fatigue (30 [26.1%] vs 16 [14.0%]; *P* = .02), abdominal pain (10 [8.7%] vs 3 [2.6%]; *P* = .047), and diarrhea (44 [38.3%] vs 27 [23.7%]; *P* = .01), which was also more frequently severe (19 [16.5%] vs 6 [5.3%]; *P* = .006), as was nausea (8 [7.0%] vs 2 [1.8%]; *P* = .052). Moreover, the probability of developing diarrhea of any grade per cycle was significantly higher with standard treatment (12-month cumulative probability, 0.41 [95% CI, 0.32-0.51] vs 0.25 [95% CI, 0.18-0.35]) (eFigures 4-6 in Supplement 2). Significantly more proteinuria was reported in the experimental arm (10 [8.8%] vs 3 [2.6%]; *P* = .049). Moreover, although lower platelet levels (32 [27.8%] vs 20 [17.5%]; *P* = .08) as well as more epistaxis (7 [6.1%] vs 15 [13.2%]; *P* = .07) were observed in the experimental arm compared with the standard arm, the difference was not statistically significant. Deaths due to toxic effects included 2 patients (with an ileal perforation and a sudden death) in the standard arm and 2 patients (with a colon perforation and a rectal perforation) in the experimental arm.

**Table 3.** Adverse Events Occurring in at Least 3% of Patients

Adverse event	Toxic effects by treatment group, No. (%)				*P* value^a^	Severe toxic effects by treatment group, No. (%)				*P* value^a^
	Standard (n = 115)		Experimental (n = 115)			Standard (n = 115)		Experimental (n = 115)		
	None	Any grade	None	Any grade		None	Grade >2	None	Grade >2	
Anemia	88 (76.5)	27 (23.5)	87 (75.7)	28 (24.3)	.88	113 (98.3)	2 (1.7)	112 (97.4)	3 (2.6)	.65
Abdominal pain	105 (91.3)	10 (8.7)	112 (97.4)	3 (2.6)	.05	110 (95.7)	5 (4.3)	113 (98.3)	2 (1.7)	.25
Diarrhea	71 (61.7)	44 (38.3)	88 (76.5)	27 (23.5)	.01	96 (83.5)	19 (16.5)	109 (94.8)	6 (5.2)	<.01
Mucositis oral	81 (70.4)	34 (29.6)	89 (77.4)	26 (22.6)	.23	109 (94.8)	6 (5.2)	111 (96.5)	4 (3.5)	.52
Nausea	71 (61.7)	44 (38.3)	71 (61.7)	44 (38.3)	>.99	107 (93.0)	8 (7.0)	113 (98.3)	2 (1.7)	.05
Vomiting	105 (91.3)	10 (8.7)	109 (94.8)	6 (5.2)	.30	114 (99.1)	1 (0.9)	114 (99.1)	1 (0.9)	>.99
Fatigue	85 (73.9)	30 (26.1)	99 (86.1)	16 (13.9)	.02	110 (95.7)	5 (4.3)	111 (96.5)	4 (3.5)	.73
Fever	108 (93.9)	7 (6.1)	107 (93.0)	8 (7.0)	.79	114 (99.1)	1 (0.9)	114 (99.1)	1 (0.9)	>.99
Pain	112 (97.4)	3 (2.6)	110 (95.7)	5 (4.3)	.47	114 (99.1)	1 (0.9)	114 (99.1)	1 (0.9)	>.99
Allergic reaction	111 (96.5)	4 (3.5)	112 (97.4)	3 (2.6)	.70	111 (96.5)	4 (3.5)	113 (98.3)	2 (1.7)	.41
Alanine aminotransferase level increased	92 (80.0)	23 (20.0)	92 (80.0)	23 (20.0)	>.99	114 (99.1)	1 (0.9)	114 (99.1)	1 (0.9)	>.99
Alkaline phosphatase level increased	92 (80.0)	23 (20.0)	93 (80.9)	22 (19.1)	.87	114 (99.1)	1 (0.9)	114 (99.1)	1 (0.9)	>.99
Neutrophil count decreased	71 (61.7)	44 (38.3)	73 (63.5)	42 (36.5)	.79	87 (75.7)	28 (24.3)	92 (80.0)	23 (20.0)	.43
Platelet count decreased	83 (72.2)	32 (27.8)	95 (82.6)	20 (17.4)	.06	114 (99.1)	1 (0.9)	113 (98.3)	2 (1.7)	.59
White blood cell count decreased	80 (69.6)	35 (30.4)	87 (75.7)	28 (24.3)	.30	107 (93.0)	8 (7.0)	110 (95.7)	5 (4.3)	.39
Peripheral neuropathy	45 (39.1)	70 (60.9)	51 (44.3)	64 (55.7)	.43	100 (87.0)	15 (13.0)	100 (87.0)	15 (13.0)	>.99
Proteinuria	112 (97.4)	3 (2.6)	105 (91.3)	10 (8.7)	.05	115 (100)	0	115 (100)	0	NA
Epistaxis	108 (93.9)	7 (6.1)	100 (87.0)	15 (13.0)	.07	115 (100)	0	115 (100)	0	NA
Voice alteration	109 (94.8)	6 (5.2)	108 (93.9)	7 (6.1)	.77	115 (100)	0	115 (100)	0	NA
Palmar-plantar erythrodysesthesia syndrome	92 (80.0)	23 (20.0)	96 (83.5)	19 (16.5)	.58	111 (96.5)	4 (3.5)	110 (95.7)	5 (4.3)	.73
Hypertension	90 (78.3)	25 (21.7)	83 (72.2)	32 (27.8)	.41	108 (93.9)	7 (6.1)	112 (97.4)	3 (2.6)	.20
Thromboembolic event	109 (94.8)	6 (5.2)	113 (98.3)	2 (1.7)	.15	112 (97.4)	3 (2.6)	113 (98.3)	2 (1.7)	.65

Abbreviation: NA, not applicable.

^a^ Calculated using the χ^2^ test.

The QOL questionnaire at baseline was completed by 203 patients (88.3%; 100 in the standard arm and 103 in the experimental arm). At least 1 later questionnaire was available for 163 patients (70.9%; 77 in the standard and 86 in the experimental arms). The global health status/QOL scores at cycles 6 and 12 were higher in patients in the experimental arm, but the difference was not statistically significant (eFigure 7 in Supplement 2). A significant improvement was shown at cycle 12 in physical functioning score (mean [SD] change from baseline, 0.65 [1.96] vs −7.41 [2.95]; *P* = .02) (eFigure 7 in Supplement 2) and constipation scores (mean [SD] change from baseline, −17.2 [3.73] vs −0.62 [4.44]; *P* = .003) (eFigure 8 in Supplement 2). Overall, 33 of 77 (42.9%) and 40 of 86 (46.5%) patients in the standard and experimental arms, respectively, reported an improvement as best QOL response in global health status (*P* = .41). Significantly more patients reported an improvement as best QOL response in physical functioning (14 of 77 [18.2%] vs 26 of 86 [30.2%]; *P* = .04) and constipation (19 of 77 [24.7%] vs 34 of 86 [39.5%]; *P* = .04) in the experimental arm (eTable 6 in Supplement 2).

## Discussion

To our knowledge, this is the first randomized clinical phase 3 trial comparing traditional concurrent and experimental sequential administration of bevacizumab in combination with standard oxaliplatin-based chemotherapy regimens in patients with mCRC. Considering the primary end point, the study failed to show any difference in ORR between treatment arms. Similarly, there was no significant difference in PFS. However, a significant advantage in OS was observed for patients in the sequential arm compared with the concurrent arm. Although OS was a secondary end point and therefore should be considered an exploratory measure of outcome, this result is of interest considering that the 2 treatment arms differed only in the bevacizumab scheduling. Given the long-term median follow-up of more than 60 months, the number of OS events appears adequately mature. The pattern of outcome of the standard arm is in keeping with the results reported in similar populations of patients with mCRC in the *RAS* unselected subgroup.^24,25,26^

The 2 treatment arms were well balanced for primary tumor site and other prognostic factors, except for *RAS* mutations, which were more represented in the experimental arm. In this regard, although *RAS* mutant status can influence intratumoral VEGF levels,^27^ the data on the predictive role of VEGF for bevacizumab-based combination treatment are largely inconsistent and, similar to *RAS*, a negative prognostic role has been highlighted.^27,28^

Furthermore, the timing of tumor response assessment was well balanced between the 2 treatment groups, and no difference in the curative rates of resection for metastases was observed. Therefore, it appears unlikely that the statistically significant OS advantage observed in the experimental arm was confounded by study management or influenced by favorable patient selection.

In addition, one of the strengths of this study was the systematic collection of data on second and subsequent therapies after progression, showing a percentage of treated patients in keeping with data reported previously.^29^ We did not observe any difference between the 2 arms in the number of patients undergoing subsequent systemic therapies; however, a higher percentage of patients in the standard arm received an anti-EGFR–containing regimen in the second line, reflecting the imbalance in distribution of patients with *RAS* wild-type alterations. Nevertheless, we did not observe any interaction between treatment outcome and *RAS* variant status, indicating that a putative reduced efficacy of anti-EGFR antibodies after antiangiogenic therapy, as reported by Bennouna et al,^30^ seems an unlikely explanation of the OS advantage observed in the experimental arm.

With respect to safety, the overall toxicity profile in both treatment arms was in agreement with that reported in previous clinical studies investigating oxaliplatin-based doublet plus bevacizumab regimens,^10,31^ and no unexpected toxic effects were observed in our study. However, the experimental arm was associated with fewer adverse events, particularly in terms of diarrhea and fatigue. Notably, the better toxicity profile observed in the experimental arm is consistent with previous findings from the phase 2 BRANCH study,^18^ exploring a similar alternative schedule of bevacizumab administration plus chemotherapy during presurgical radiotherapy in high-risk patients with locally advanced rectal cancer.

As a consequence of the more favorable safety profile, the experimental arm was also associated with better treatment compliance and health-related QOL. Indeed, more patients completed treatment induction and received maintenance therapy in the experimental arm, and fewer patients in this arm needed treatment delay and dose reduction compared with the standard arm. Likewise, a clear trend to better global health status/QOL and a significant improvement of physical functioning and constipation were reported in the experimental arm. Although QOL data yield complementary information, they add value to our study, being associated with real clinical benefit and considering that QOL reporting was not available in most recently published phase 3 trials in mCRC.^32^

In recent years, an emerging new concept explored in preclinical models suggests that the mechanism behind improvement in survival with antiangiogenic therapy may be related—in addition to tumor size reduction—to off-tumor targets.^33^ Antiangiogenic drugs may improve chemotoxic tolerance by modulating the function of various tissues and organs through systemic alteration of the vasculature, thus preventing cancer-associated systemic syndromes.^33^ In this regard, our safety data are consistent with preclinical evidence showing a significant reduction of chemotoxic effects and a superior survival effect with the sequential delivery of antiangiogenic drugs and chemotherapy compared with concomitant administration.^34^

On the other hand, several studies have suggested that antiangiogenic drugs may act as chemosensitizing agents by blunting the protumorigenic and prometastatic effects of the host response to conventional chemotherapy.^35^ Importantly, a recent preclinical study^36^ showed that scheduling of bevacizumab administration 3 days before chemotherapy improved antitumor efficacy and reduced metastatic spread compared with concomitant administration. These additional mechanisms could explain how antiangiogenic agents can improve efficacy of chemotherapy beyond vessel normalization and suggest that the potential synergistic effect of antiangiogenesis in combination treatment may not be determined only by the improvement of drug delivery. Several preclinical and clinical studies with radiolabeled drugs^37^ have questioned the postulated mechanism of improved drug delivery as a consequence of anti-VEGF treatment. Moreover, tumor uptake of radiolabeled drugs may not be a good predictor of clinical benefit.^38^

The importance of vascular normalization and of bevacizumab scheduling to optimize the efficacy of treatment has been reported with several combination therapies.^39^ Compelling recent findings also show that vascular normalization might favor an immune-supportive tumor microenvironment, suggesting the combination of bevacizumab with immunotherapy as an attractive anticancer therapeutic approach.^40^ Interestingly, mathematical models suggest that antiangiogenic treatment may improve immunotherapy when the 2 treatments are administered sequentially.^41^ Overall, these findings suggest that host response and off-tumor targets may be more relevant in the sequential schedule and could explain, at least in part, the discordance between ORR and survival outcome observed in our trial.

### Limitations

Our study has some limitations. Considering that the accuracy of conventional evaluation of tumor response by RECIST criteria to bevacizumab combination treatment has been questioned,^42,43^ we acknowledge that our choice of ORR as primary end point may have been inadequate. Indeed, several lines of evidence suggest that alternative morphological criteria such as the MD Anderson criteria are better correlated with long-term outcome.^43^ We are also aware that balancing out unmeasurable differences in patient outcome through randomization is not always achieved with confidence in trials with a relatively small sample size, as in our study. In addition, we cannot definitively rule out that a different interval between bevacizumab and chemotherapy could improve the efficacy of combination treatment.^44^

## Conclusions

The results of this randomized clinical trial failed to show an improvement of the primary end point ORR from the sequential administration of bevacizumab in combination with standard oxaliplatin-based regimens in unselected patients with *RAS* mutations and mCRC. However, although hypothesis generating, the OS advantage, fewer adverse effects, and better health-related QOL observed in the sequential scheduling of bevacizumab administration compared with concomitant administration warrant consideration for additional clinical studies. Indeed, sequential bevacizumab administration plus chemotherapy might be relevant to optimize therapeutic efficacy and to explore antiangiogenic combination treatments with an innovative perspective.

## Notes

Supplement 1. Trial Protocol

Supplement 2. **eFigure 1.** Scatterplot of the Timing of the Evaluation of Objective Response by Study Arm During Chemotherapy Treatment (12 and 24 Weeks After Randomization) **eFigure 2.** Forest Plot of Progression-Free Survival by Patient Subgroups **eFigure 3.** Forest Plot of Overall Survival by Patient Subgroups **eFigure 4.** Analysis of Diarrhea Occurrence **eFigure 5.** Analysis of Nausea Occurrence **eFigure 6.** Analysis of Fatigue Occurrence **eFigure 7.** Mean Changes From Baseline by Study Arm in EORTC QLQ-C30 Functional Scales **eFigure 8.** Mean Changes From Baseline by Study Arm in EORTCQLQ-C30 Symptom Items **eTable 1.** Compliance With Planned Treatment **eTable 2.** Best Overall Response to Treatment by Subgroups *RAS* Status **eTable 3.** Metastases-Resected Patients **eTable 4.** Postprogression Therapies According to Treatment Arm **eTable 5.** Worst per Patient Adverse Events Reported During Treatment, Classified According to the CTCAE, Version 4.0 **eTable 6.** Distribution of Best QOL Response From Baseline With EORTC QLQ-C30 Global Health Status, Functional Scales, and Symptom Items

Supplement 3. Data Sharing Statement
