# Supplementary material for: Effect of Bevacizumab in Combination With Standard Oxaliplatin-Based Regimens in Patients With Metastatic Colorectal Cancer: A Randomized Clinical Trial
Source: JAMA Netw Open. 2021 Jul 26;4(7):e2118475. doi: 10.1001/jamanetworkopen.2021.18475 (PMC8314140; doi:10.1001/jamanetworkopen.2021.18475)
Supplement: Supplement 1. — Trial Protocol [file jamanetwopen-e2118475-s001.pdf]

EUDRACT NUMBER: 2011-004997-27***; ClinicalTrials.gov number***: NCT01718873

Randomized phase 3 study on the optimization of the combination of bevacizumab with FOLFOX/OXXEL in the treatment of patients with metastatic colorectal cancer.

Nickname: OBELICS (Optimization of BEvacizumab scheduLIng within Chemotherapy Scheme)

No-profit Sponsor:
National Cancer Institute of Naples (NCI)

SYNOPSIS

Study coordinator (Principal Investigator and Chief Scientist of Study) :

Dr. A. Avallone	Medical Abdominal Oncology, NCI of Naples

Principal Investigators (Co-Investigators):

Prof. R.V. Iaffaioli	Medical Abdominal Oncology, NCI of Naples
Dr. A. Budillon	Experimental Pharmacology, NCI of Naples
Dr. F. Perrone	Clinical Trial Unit, NCI of Naples
Dr. S. Lastoria	Nuclear Medicine and Metabolic Therapy, NCI of Naples
Prof. C.Gallo	Medical Statistics, Second University of Naples
To determinate	Other Institutions

Scientific Board:

R.Casaretti, G.Nasti	Medical Abdominal Oncology, NCI of Naples
G.Romano, P.Delrio, F.Izzo	Abdominal Cancer Surgery, NCI of Naples
E. Di Gennaro, A.Leone	Experimental Pharmacology, NCI of Naples
A.Petrillo, O.Catalano	Radiology, NCI of Naples
L.Aloj, C.Caracò	Nuclear Medicine and Metabolic Therapy, NCI of Naples
F.Tatangelo, G.Botti,	Pathological Anatomy, NCI of Naples
S.Cavalcanti, L.Nitch, D.Grilli	Clinical Pathology, NCI of Naples
M.C.Piccirillo, F.Perrone	Clinical Trial Unit, NCI of Naples

Secretary and Coordinating Center of Clinical Study:

M.C.Piccirillo, P.Giordano,M.Florio	Clinical Trial Unit, NCI of Naples

Secretary and Coordinating of Biological Studies:

A.Budillon, E. Di Gennaro, A.Leone, A. De Caro	Experimental Pharmacology, NCI of Naples

Coordinating Ethics Committee: National Cancer Institute of Napoli

Version n.1 0 of October 06, 2011

STUDY SYNOPSIS

Rationale

Although the approval of bevacizumab has changed clinical practice in the treatment of colorectal cancer (CRC), the efficacy demonstrated by this anti-vascular endothelial growth factor (VEGF) in combination with chemotherapy, has thus far been rather modest. Therefore, it is crucial to develop more effective ways to combine anti-angiogenic drugs with chemotherapeutic treatments, and to identify valid predictive biomarkers of benefit anti-angiogenic approach, in order to avoid ineffective and expensive therapies in non-responder patients.

Increasing evidences show that, in combination with chemo e/o radiotherapy, anti-angiogenic schedule could be relevant to optimize the feasibility and efficacy of radio- and chemotherapy.

Preliminary results of BRANCH study, a phase II study conducted at the National Cancer Institute of Naples, in patients with locally advanced rectal cancer, have shown a reduced toxicity and an increased efficacy of experimental “timing” of bevacizumab administration, 4 days before chemo-radiotherapy, compared with the standard administration of bevacizumab and chemo-radiotherapy in combination, on the first day.

This experimental schedule of bevacizumab induced a lower incidence of grade 3-4 neutropenia, and higher rate of complete tumor regression rate (TRG1) than reported with the traditional concurrence administration of bevacizumab.

There is an unmet need for pharmacodynamic and predictive biomarkers of benefit for anti-angiogenic drugs.

In cancer patients treated with anti-angiogenic therapy, the ability to noninvasively measure single nucleotide polymorphisms (SNPs) of VEGF gene, the levels (baseline and during treatment) of circulating endothelial precursors cells (CECs) and their progenitors (CEPs) count together with a broad profile of cytokines and angiogenic factors could help to select the patients most likely to benefit from these high-cost therapies, and to identify possible mechanisms of resistance and consequently new targets for improving anti-angiogenic therapy.

Multiplex technologies offer a noninvasive, easy and convenient method of simultaneously assessing a much larger number of biologically relevant citochine and angiogenic factor from small plasma volumes. Moreover, the high stability of miRNA in the plasma of patients with cancer and their correlation with the expression in the tumor, suggest the possibility to identify innovative predictive biomarkers of benefit for anti-angiogenic therapy.

Pursuing imaging and circulating biomarkers shortly after treatment initiation might be a fruitful approach, as many of the biomarker changes occur rapidily after the onset of therapy. The ability to identify tumor-specific changes rapidily after treatment may allow tailoring of therapy to those patients most likely to benefit, and early discontinuation of an ineffective therapy in other.

In this study we will compare the traditional concurrent administration of bevacizumab in combination with chemotherapy, with an experimental schedule, defined on the basis of "normalization hypothesis", in which bevacizumab will be given 4 days before chemotherapy.

National Cancer Institute of Naples
www.usc-intnapoli.net
Tel 0815903571 - fax 0817702938

Study Objectives

Primary Objective

- To assess whether an experimental schedule of bevacizumab, given in sequence instead of in combination with oxaliplatin regimen (mFOLFOX/mOXXEL), can improve treatment activity (in terms of objective response rate) in patients with metastatic colorectal cancer

Secondary Objectives

- To evaluate the impact of the experimental schedule on:

- Progression free survival (PFS)
- Overall survival (OS).
- Toxicity.
- Quality of life.

- To evaluate prognostic and predictive value of :

- Circulating endothelial cells (CEC) and Circulating endothelial precursor cells (CEP) count on patient blood samples.
- Cytokine and circulating angiogenic factors plasma levels on patient blood samples.
- Single nucleotide polymorphisms (SNPs) of VEGF on patient blood samples.
- WBC counts at 24 hours after the 1st administration of bevacizumab on patient blood samples.
- microRNAs (miRNAs) on patient blood samples.
- Change of tumor metabolic volume, at 11 days after the start of the first cycle of chemotherapy in both arms (15 days after the first administration of bevacizumab in the experimental arm) evaluated by [18F]Fluorodeoxyglucose positron emission tomography (FDG-PET).

Study Design

This is a prospective, multicentre, open label, randomised phase 3 trial comparing two treatment strategies. Eligible patients will be randomly assigned with 1:1 ratio to the following arms :

- **Standard arm:** bevacizumab in combination with modified FOLFOX-6 regimen (mFOLFOX-6) or modified OXXEL regimen (mOXXEL), in which bevacizumab will be administered before oxaliplatin, on day 1 of each cycle.

- **Experimental arm:** bevacizumab in sequence with mFOLFOX-6 or mOXXEL regimen, in which bevacizumab will be administered 4 days before oxaliplatin of each cycle.

In both arms of treatment, the patients without progression after 12 cycles (24 weeks) of treatment will stop chemotherapy and will continue bevacizumab (7,5mg/kg every 3 weeks) until progression, unacceptable toxicity or patient's choice to stop.

Oxaliplatin regimen (mFOLFOX/mOXXEL) will be chosen according to centre policy at the beginning of the study.

Primary end point is objective response rate (RR) according to RECIST criteria.

The sample size of study depends on the expected response rate for the control arm; it varies as a function of the proportion of patients enrolled in first and in second line of treatment. Assuming 40% as response rate for the first line of therapy and 20% for the second line, the sample size is calculated within three different expected response rate: 35% (75% as first line and 25% as second line), 30% (50% and 50% respectively) and 25% (25% and 75% respectively). In the following table, the expected sample size is reported, under the three hypothesized conditions, fixing an odds ratio of 2,25, 80% statistical power and 2-sided alpha error of 0.05. We have also reported the auspicated response rates, in the experimental arm, and the corresponding relative risks, ranging 1.6 to 1.7. As expected, odds ratio represents an overestimation of relative risk, and the extent of overestimation increases with increasing the expected response rate.

**% expected response in standard arm**	**# patients**	**% expected response in experimental arm**	**Relative risk (experimental *vs* standard)**
**35**	**201**	**54,8%**	**1,57**
**30**	**209**	**49,1%**	**1,63**
**25**	**225**	**42,9%**	**1,71**

Therefore, a sample size of 220-230 patients will allow reliable assessment in all plausible case-mix conditions.

With a sample size of 225 patients and a speed of accrual of 15 patients/month, the study will achieve 163 events, necessary for identifying a 5 months PFS prolongation, corresponding to a 0.64 hazard ratio, with an expected PFS of 9 months in the standard arm, and a 2-sided alpha error of 0.05.

Inclusion criteria

- Histological diagnosis of adenocarcinoma of the colon or rectum
- Stage IV of disease
- Presence at least one measurable target lesion (according to the RECIST criteria) not previously treated with radiotherapy.
- Ages > 18 and < 75 years
- ECOG performance status 0 or 1 at study entry
- Life expectancy > 3 months
- Adequate recovery from recent surgery (at least 28 days after a major surgery or a biopsy).
- Effective contraception both for male and female patients, if the risk of conception exists.
- Signed informed consent.

Exclusion Criteria

- More than one line of treatment for metastatic disease.

- Previous treatment with bevacizumab or oxaliplatin (a previous treatment with fluoropirymidines, folic acid, irinotecan or cetuximab is allowed).
- Presence of primary colorectal cancer that produces stenosis or full-thickness wall infiltration.
- Regular use of NSAID or aspirin (more than 325 mg/die).
- Bleeding diathesis or pre-existing coagulopathy.
- Use of anticoagulants at therapeutic dose.
- Known or suspected brain metastases (determined exclusively in the presence of at least one clinical symptom)
- Neutrophils < 2000/mm^3 or platelets < 100.000/ mm^3 or haemoglobin <9 gr/dl.
- Creatinine >1.5 time the upper normal limit (UNL).
- GOT and/or GPT > 2.5 time the UNL and/or bilirubin >1.5 time the UNL in absence of liver metastasis.
- GOT and/or GPT > 5 time the UNL and/or bilirubin > 3 time the UNL in presence of liver metastasis
- Any concurrent malignancy other than non-melanomatous skin cancer, or carcinoma in situ of the cervix. Patients with a previous malignancy but without evidence of disease for 5 years will be allowed to enter the trial.
- Congestive heart failure, recent ischemic coronary disease (in the last 12 months), uncontrolled arrhythmia.
- Uncontrolled hypertension.
- Active or uncontrolled infection.
- A serious uncontrolled medical disorder that in the opinion of the investigator would impair the ability of the subject to receive protocol therapy.
- Pregnancy (absence to be confirmed by B-HCG test) or lactation period.
- History or current evidence on physical examination of central nervous system disease or Peripheral Neuropathy > Grade 1 (CTCAE v. 4.0)
- Unable to comply with follow-up

Treatment Plan

Arms of study:

- **Standard Arm:** Bevacizumab (5mg/kg) and mFOLFOX-6 regimen (oxaliplatin 85 mg/m2 i.v. infusion on day 1 followed by levo-folinic acid 200 mg/m2 i.v. infusion followed by i.v bolus. 5-fluorouracil 400 mg/m2, and a 46-hour i.v. infusion of 5-fluorouracil 2400 mg/m2) or mOXXEL regimen (oxaliplatin 85 mg/m2 i.v. infusion on day 1 plus oral capecitabine 1000 mg/m2 twice daily on days 1 to 10) every 2 weeks for 12 cycles (24 weeks); Bevacizumab will be administered as 20- to-30 minute intravenous infusion before oxaliplatin on day 1 of each cycle of treatment.

- **Experimental Arm**: Bevacizumab (5mg/kg) on day 1 and mFOLFOX-6 or mOXXEL regimen (at same doses used in the standard arm) after 4 days every 2 weeks for 12 cycles (24 weeks)

In both arms of treatment the patients who will be progression free after 12 cycles (24 weeks) of treatment will stop chemotherapy and will continue bevacizumab (7,5mg/kg every 3 weeks) until progression, unacceptable toxicity or patients chose to stop.

Baseline Procedures

Before any study procedure:
- Signed informed consent

Within 14 days prior to registration:
- Complete medical history, physical examination, evaluation of ECOG PS and vital signs (included blood pressure).
- Evaluation of quality of life (QoL) by EORTC QLQ-C30 questonnaire.
- Blood count and biochemistry analyses (included B-HCG test for the women of childbearing age)
- CEA and CA19.9
- CEC and CEP counts, evaluation of cytochine and circulating angiogenic factors plasma levels, evaluation of SNPs of VEGF and miRNAs.

Within 21 days prior to registration:
- ECG
- Thoracic and abdomen computer tomography (CT) scan.
- 18FDG-PET for the tumor metabolic volume assessment.
- Other tests may be performed at the Researcher's discretion.

During the treatment with bevacizumab and chemotherapy

- Physical examination and vital signs (included blood pressure) at 1st day of every cycle of treatment.
- Blood pressure evaluation immediately after the administration of bevacizumab (in the hospital) and 24 and 48 hours after the administration of bevacizumab of every cycle of treatment,in both arms of treatment (even at home)
- WBC counts 24 hours after the 1st administration of bevacizumab in both arms of treatment
- Blood count every week and at 1st day of every cycle of treatment
- Biochemistry and Urinalysis 1st day of every cycle of treatment.
- Bloods samples for CEC and CEP counts, evaluation of cytochine and circulating angiogenic factors plasma levels and evaluation of miRNAs will be collected at 15th day after starting treatment (the day in which starting 2nd cycle of treatment) before the administration of bevacizumab. Further bloods samples must be collected at 12th and 24th week (at 1st and 2nd response evaluation).
- CEA and CA19.9, ECG, QoL and thoracic and abdomen CT scan at 12th and 24th week after starting treatment
- Tumor metabolic volume assessment by 18FDG-PET at 11th +/- 2 days after starting chemotherapy treatment in both arms (15 days after the first administration of bevacizumab in the experimental arm; in all cases the PET scan must be done before the 2nd administration of bevacizumab).
- Anyone tests that the Researcher will believe clinically indicated

During the follow up after the stop of chemotherapy and the treatment with bevacizumab

- Physical examination, vital signs (included blood pressure), blood count and biochemistry every three weeks, before every administration of bevacizumab. .
- CEA and Ca19.9, ECG, QoL and thoracic and abdomen CT scan every three months.
- Bloods samples for CEC and CEP counts, evaluation of cytochine and circulating angiogenic factors plasma levels and miRNA, will be collected at progression of disease
- Anyone tests that the Researcher will believe clinically indicated

During the follow-up after the progression disease

- Physical examination, evaluation of vital signs and registration of any medical treatment every three months.

Toxicity evaluation criteria

Toxicity will be graded according to the Common Terminology Criteria for Adverse Events (CTCAE) of the National Cancer Institute, version 4.0, June 14, 2010 (see http://evs.nci.nih.gov/ftp1/CTCAE/CTCAE_4.03_ 2010-06-14_QuickReference_ 5x7.pdf).

Response evaluation criteria

Response evaluation will be performed at weeks 12 and 24, after the onset of the trial, and every 3 months thereafter, until disease progression, with the following examination :

-CT scan of chest, abdomen and pelvis
-CEA, CA 19.9
-Any other tests positive results during staging

Objective response will be categorized according to Response Evaluation Criteria in Solid Tumors (RECIST) v. 1.1. An independent blinded central review of radiologic examinations will be performed.

Registration and data collection procedures

Web-based procedures for registration, randomization and data collection will be centralized through the Clinical Trials Unit of the NCI of Naples (http://www.usc-intnapoli.net). Biological data collection will be done centrally at the Experimental Pharmacology Unit of the NCI of Naples. Randomization will be performed with a minimization procedure that will account for the following parameters as strata: center, performance status (0 vs 1), previous chemotherapy for advanced disease (yes vs no) and number of metastatic sites (1 vs more).

Statistical analysis

All analyses will be performed according to an intention to treat strategy

Objective Response rate (RR)

RR is defined as the number of complete plus partial response divided by the total of patients enrolled in each comparison arm. Objective RR will be described by 2x2 contingency tables and statistical significance of the possible difference will be estimated by chi-square test. The difference between RR in the two arms will be estimated with 95% confidence interval.

Progression Free Survival (PFS)

PFS is defined as the time from randomization to the date of progression, the date of death without progression or the date of the last follow-up information available (for patients losT before the trial end date), whichever occurred first. Curves will be drawn with the Kaplan-Meier product-limit method. Statistical significance will be calculated by a model of multivariable analysis considering stratification factors as covariates.

Overall Survival (OS)

OS is defined as the time from randomization to the date of death or the date of termination of the trial (for patients alive at the time end of the study), or the date of the last follow-up information available (for patients loss before the trial end date). Curves will be drawn with the Kaplan-Meier product-limit method. Statistical significance will be calculated by a model of multivariable analysis considering stratification factors as covariates.

Toxicity

For each patient and type of toxicity, the worst degree suffered during the treatment will be describe. Patients who will have not received assigned treatment will be excluded. Statistical analysis will be performed by contingency tables and statistical significance of the possible differences between the treatment groups will be calculated with a linear permutation test accounting for ordinal nature of data (linear rank test).

Administrative aspects

The trial is a multicentre, independent investigator-initiated study; it is supported by a grant of the Ministry of Health (RF-2009-1539464). NCI of Naples will take out insurance coverage for trial participants.

Drugs on study

All drugs are approved by the Italian National Health Service for the treatment of metastatic colorectal cancer patients.
