# Supplementary material for: Effect of Bevacizumab in Combination With Standard Oxaliplatin-Based Regimens in Patients With Metastatic Colorectal Cancer: A Randomized Clinical Trial
Source: JAMA Netw Open. 2021 Jul 26;4(7):e2118475. doi: 10.1001/jamanetworkopen.2021.18475 (PMC8314140; doi:10.1001/jamanetworkopen.2021.18475)
Supplement: Supplement 2. — eFigure 1. Scatterplot of the Timing of the Evaluation of Objective Response by Study Arm During Chemotherapy Treatment (12 and 24 Weeks After Randomization) eFigure 2. Forest Plot of Progression-Free Survival by Patient Subgroups eFigure 3. Forest Plot of Overall Survival by Patient Subgroups eFigure 4. Analysis of Diarrhea Occurrence eFigure 5. Analysis of Nausea Occurrence eFigure 6. Analysis of Fatigue Occurrence eFigure 7. Mean Changes From Baseline by Study Arm in EORTC QLQ-C30 Functional Scales eFigure 8. Mean Changes From Baseline by Study Arm in EORTCQLQ-C30 Symptom Items eTable 1. Compliance With Planned Treatment eTable 2. Best Overall Response to Treatment by Subgroups RAS Status eTable 3. Metastases-Resected Patients eTable 4. Postprogression Therapies According to Treatment Arm eTable 5. Worst per Patient Adverse Events Reported During Treatment, Classified According to the CTCAE, Version 4.0 eTable 6. Distribution of Best QOL Response From Baseline With EORTC QLQ-C30 Global Health Status, Functional Scales, and Symptom Items [file jamanetwopen-e2118475-s002.pdf]

Supplementary Online Content

Avallone A, Piccirillo MC, Nasti G, et al. Effect of bevacizumab in combination with standard oxaliplatin-based regimens in patients with metastatic colorectal cancer: a randomized clinical trial. *JAMA Netw Open*. 2021;4(7):e2118475. doi:10.1001/jamanetworkopen.2021.18475

eFigure 1. Scatterplot of the Timing of the Evaluation of Objective Response by Study Arm During Chemotherapy Treatment (12 and 24 Weeks After Randomization)

eFigure 2. Forest Plot of Progression-Free Survival by Patient Subgroups

eFigure 3. Forest Plot of Overall Survival by Patient Subgroups

eFigure 4. Analysis of Diarrhea Occurrence

eFigure 5. Analysis of Nausea Occurrence

eFigure 6. Analysis of Fatigue Occurrence

***e*Figure 7.** Mean Changes From Baseline by Study Arm in EORTC QLQ-C30 Functional Scales

***e*Figure 8.** Mean Changes From Baseline by Study Arm in EORTCQLQ-C30 Symptom Items

eTable 1. Compliance With Planned Treatment

eTable 2. Best Overall Response to Treatment by Subgroups *RAS* Status

eTable 3. Metastases-Resected Patients

eTable 4. Postprogression Therapies According to Treatment Arm

eTable 5. Worst per Patient Adverse Events Reported During Treatment, Classified According to the CTCAE, Version 4.0

eTable 6. Distribution of Best QOL Response From Baseline With EORTC QLQ-C30 Global Health Status, Functional Scales, and Symptom Items

This supplementary material has been provided by the authors to give readers additional information about their work.

© 2021 Avallone A et al. *JAMA Network Open*.

eFigure 1. Scatterplot of the Timing of the Evaluation of Objective Response by Study Arm During Chemotherapy Treatment (12 and 24 Weeks After Randomization)

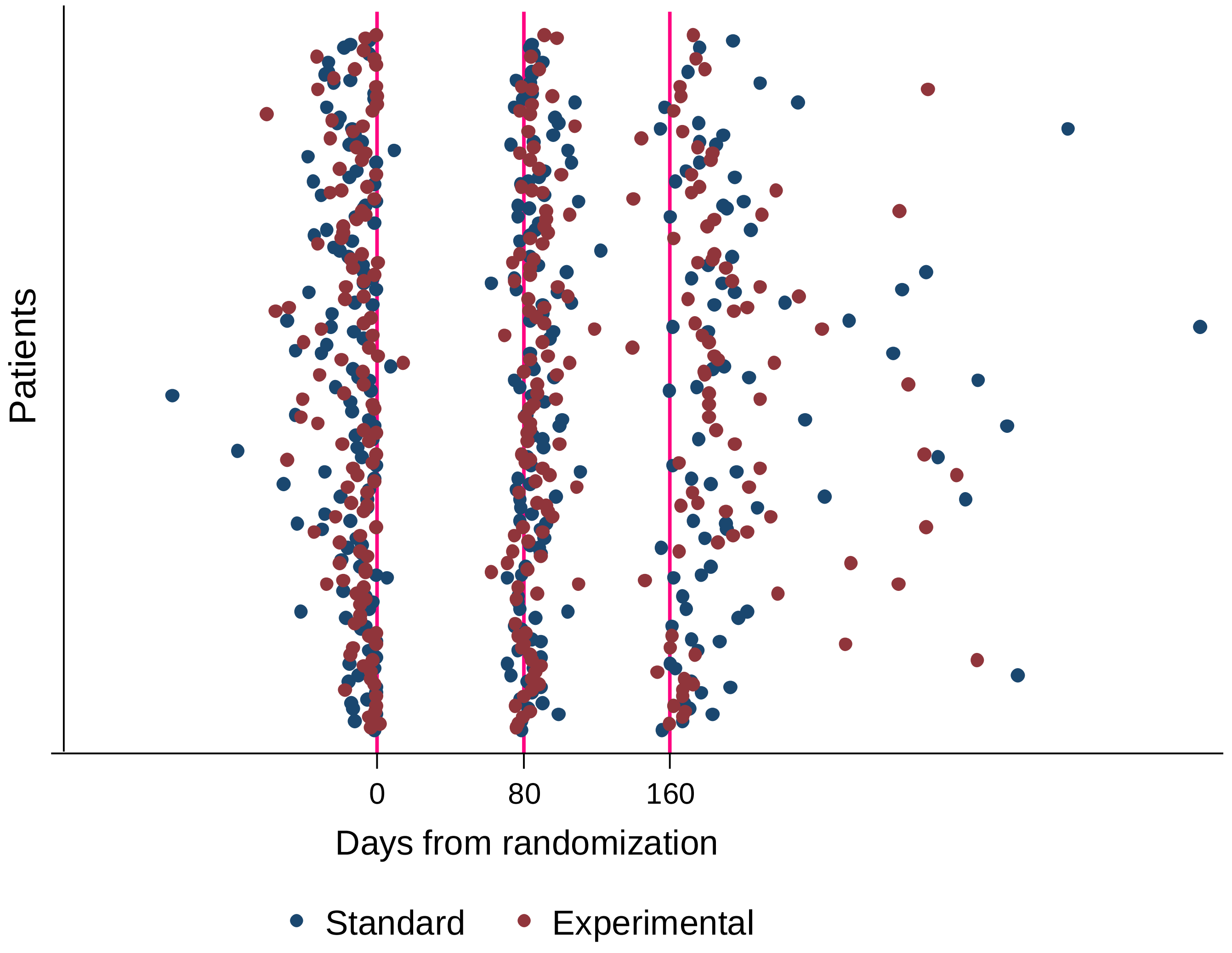

© 2021 Avallone A et al. *JAMA Network Open*.

eFigure 2. Forest Plot of Progression-Free Survival by Patient Subgroups

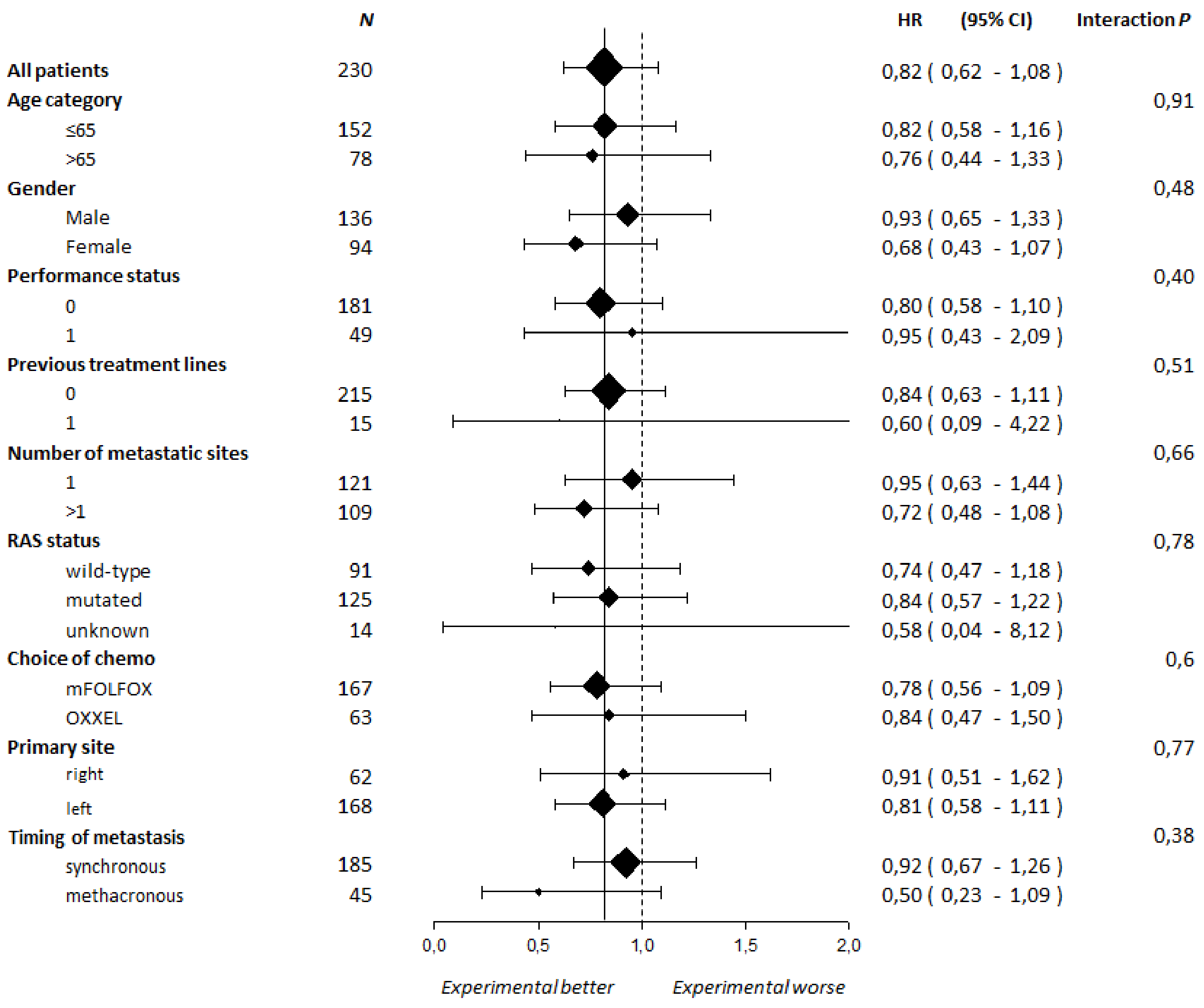

The size of the diamonds is proportional to the size of the corresponding subgroup.

© 2021 Avallone A et al. *JAMA Network Open*.

eFigure 3. Forest Plot of Overall Survival by Patient Subgroups

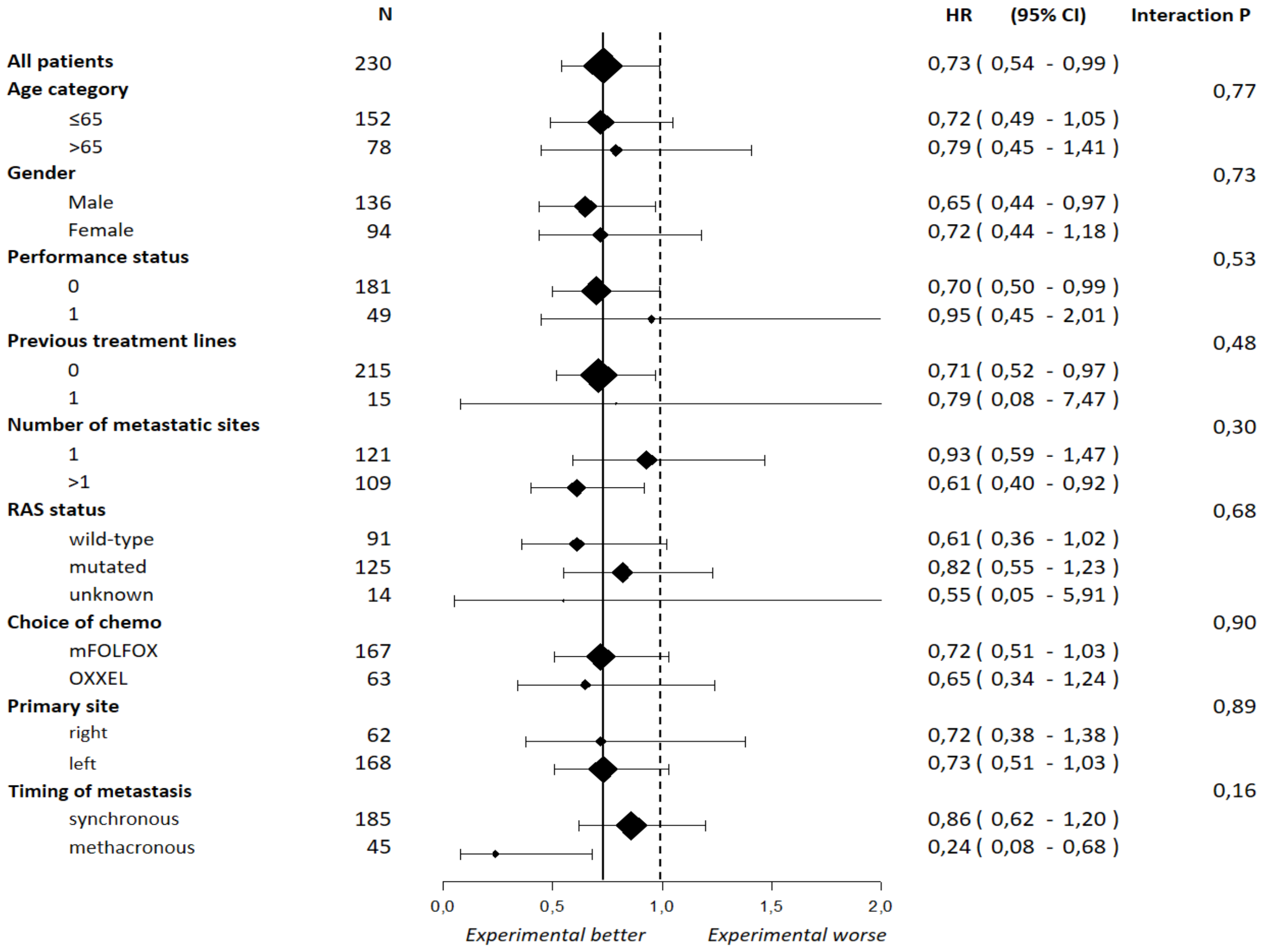

The size of the diamonds is proportional to the size of the corresponding subgroup.

© 2021 Avallone A et al. *JAMA Network Open*.

eFigure 4. Analysis of Diarrhea Occurrence

Panel A. Butterfly chart of percentage incidence of diarrhea per cycle; Panel B. Probability of developing any grade diarrhea per cycle; Panel C. Probability of developing severe grade diarrhea per cycle

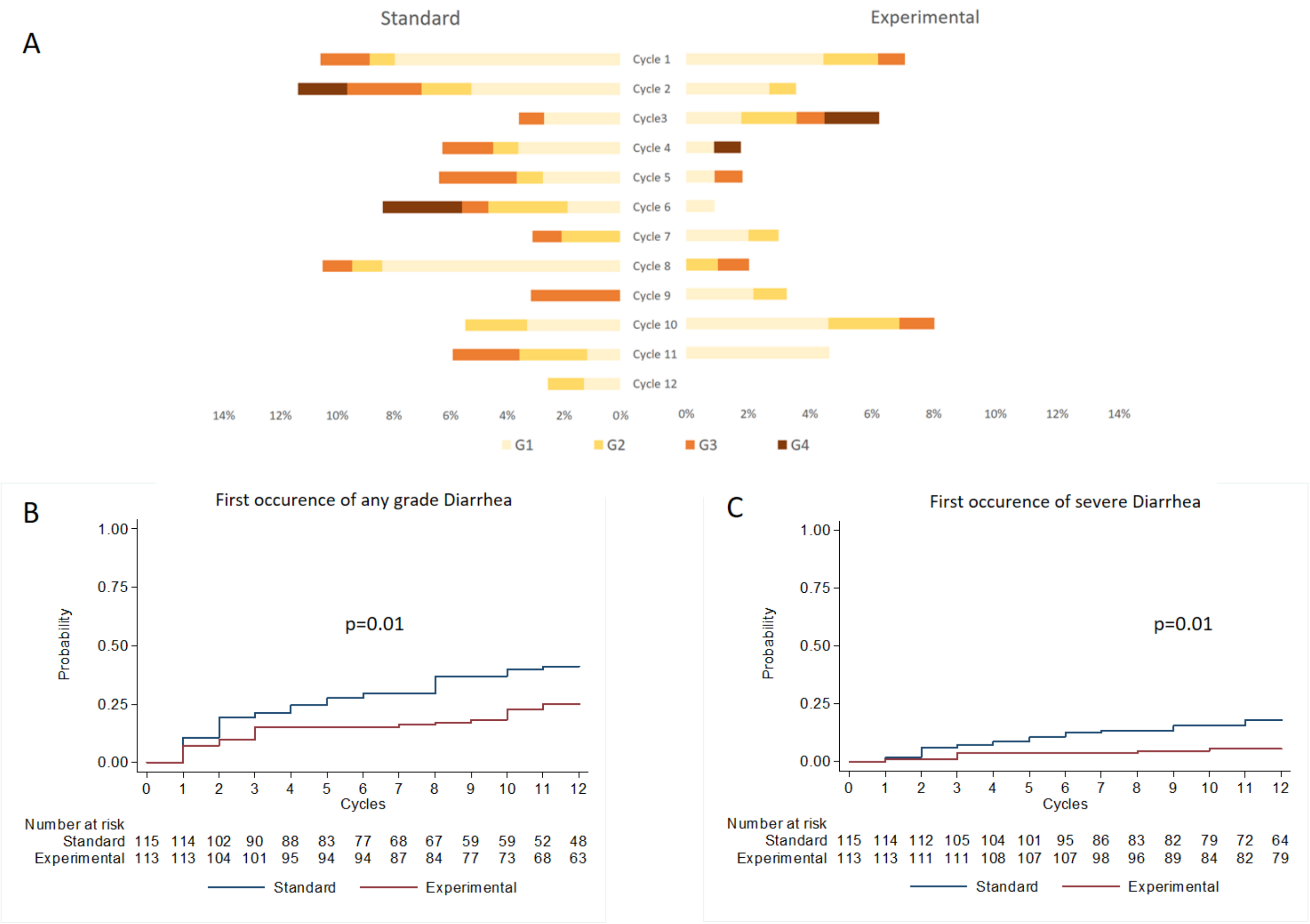

© 2021 Avallone A et al. *JAMA Network Open*.

eFigure 5. Analysis of Nausea Occurrence

Panel A. Butterfly chart of percentage incidence of nausea per cycle; Panel B. Probability of developing any grade nausea per cycle; Panel C. Probability of developing severe grade nausea per cycle

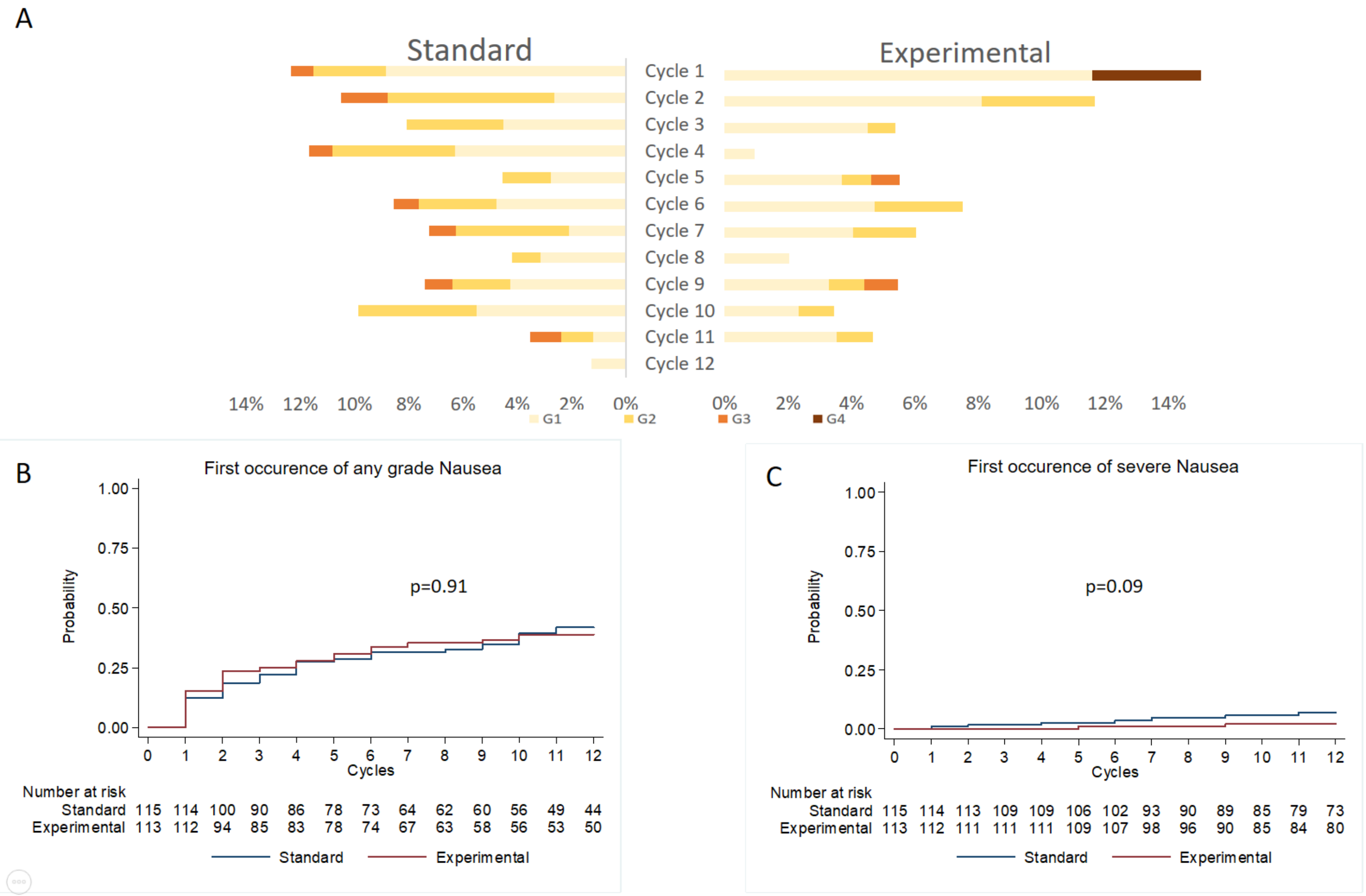

© 2021 Avallone A et al. *JAMA Network Open*.

eFigure 6. Analysis of Fatigue Occurrence

Panel A. Butterfly chart of percentage incidence of fatigue per cycle; Panel B. Probability of developing any grade fatigue per cycle; Panel C. Probability of developing severe grade fatigue per cycle

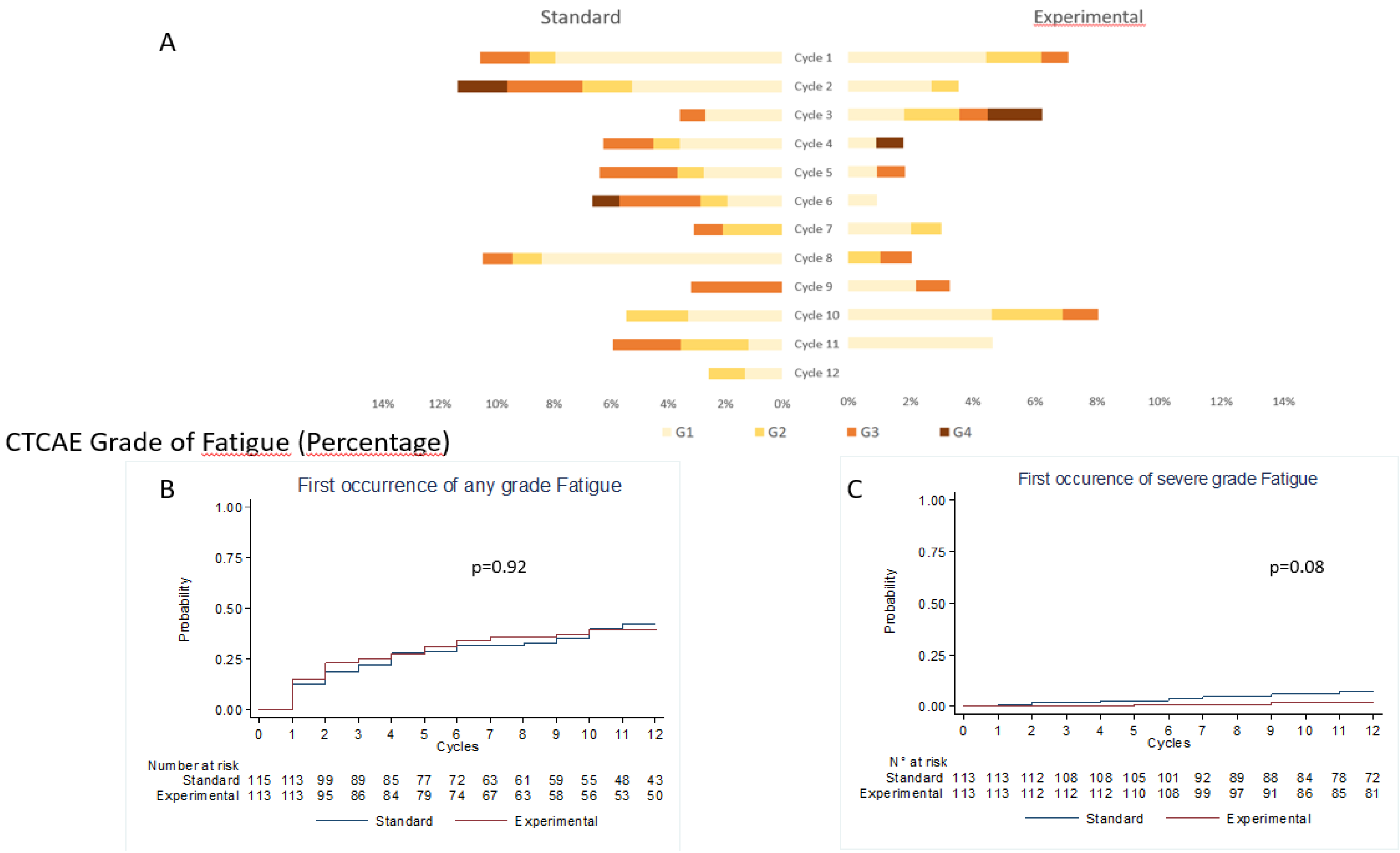

© 2021 Avallone A et al. *JAMA Network Open*.

***e*Figure 7.** Mean Changes From Baseline by Study Arm in EORTC QLQ-C30 Functional Scales

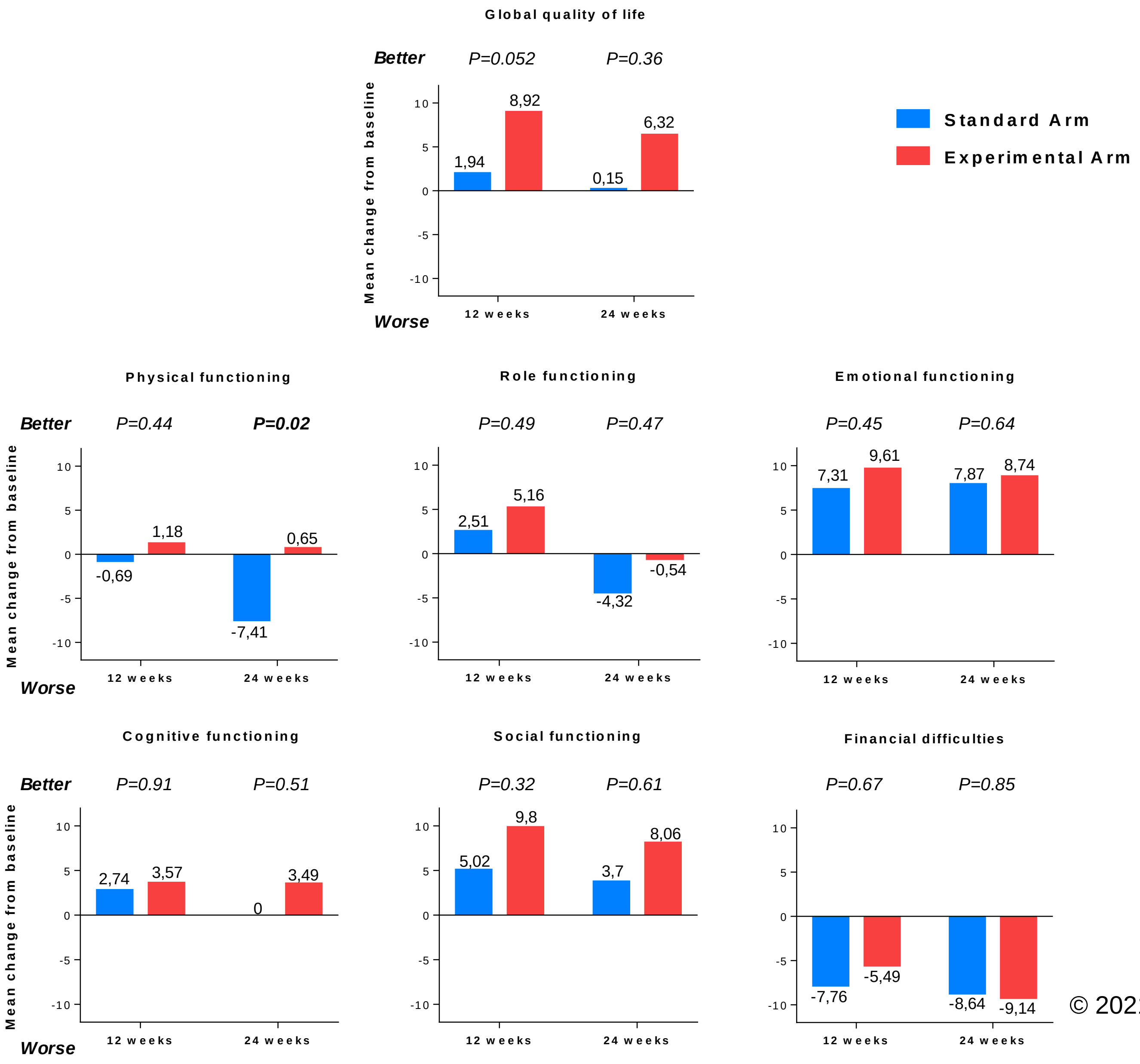

© 2021 Avallone A et al. *JAMA Network Open*.

***e*Figure 8.** Mean Changes From Baseline by Study Arm in EORTCQLQ-C30 Symptom Items

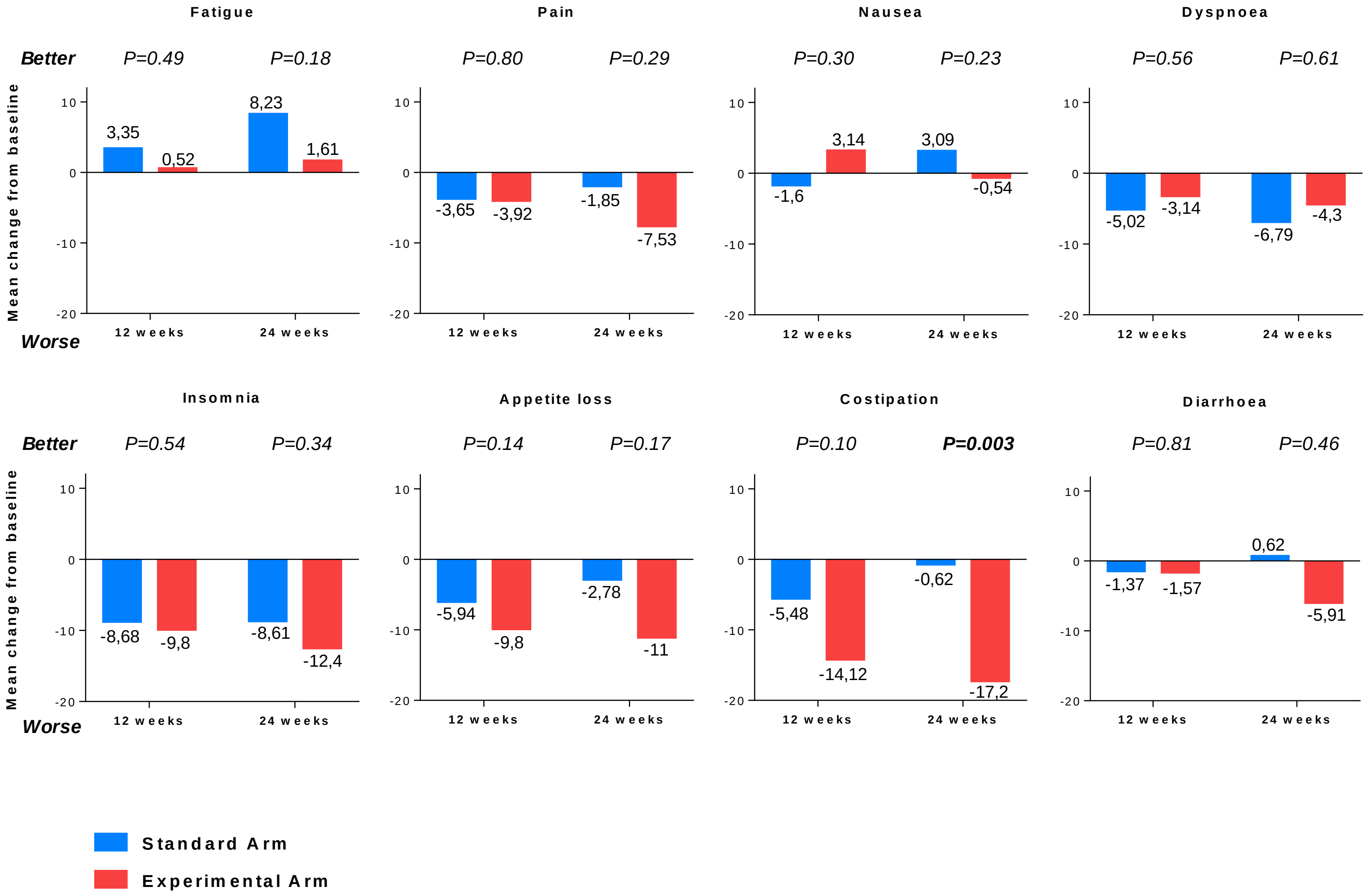

© 2021 Avallone A et al. *JAMA Network Open*.

eTable 1. Compliance With Planned Treatment

	**Standard Arm (n 115)**	**Experimental Arm (n 114)**
**No. of chemo/beva cycles,** median (IQR)	12 (10-12)	12 (10-12)
**Patients needing a dose reduction,** n (%)	14 (12.2%)	6 (5.3%)
**Patients needing a treatment delay,** n (%)	30 (26.1%)	22 (19.3%)
**Cause of chemo/beva treatment interruption,** n (%)		
Completion	78 (67.8%)	83 (72.8%)
Progression or death	11 (9.6%)	13 (11.4%)
Toxicity or refusal	12 (10.4%)	5 (4.4%)
Surgery	10 (8.7%)	12 (10.5%)
Medical decision	4 (3.5%)	-
**Patients starting maintenance,** n (%)	44 (38.3%)	52 (45.2%)
Bevacizumab + 5FUFA/capecitabine	38 (33.0%)	46 (40.0%)
Bevacizumab	6 (5.2%)	6 (5.3%)
**No. of maintenance cycles,** median (IQR)	4 (2-6)	6 (4-8)
**Cause of maintenance treatment interruption,** n (%)		
Progression or death	22 (19.1%)	27 (23.7%)
Toxicity or refusal	4 (3.5%)	3 (2.6%)
Surgery	12 (10.4%)	10 (8.8%)
Medical decision	7 (6.1%)	14 (12.3%)

© 2021 Avallone A et al. *JAMA Network Open*.

eTable 2. Best Overall Response to Treatment by Subgroups *RAS* Status

	Standard	Experimental
**RECIST response**		
**Response rate in RAS wild type (N=91)**		
Responder (CR+PR) No. (%)	35 (64.8%)	26 (70.3%)
Not Responder (SD+PD) No. (%)	19 (35.2%)	11 (29.7%)
**Response rate in RAS mutant (N=125)**		
Responder (CR+PR) No. (%)	29 (53.7%)	37 (52.11%)
Not Responder (SD+PD) No. (%)	25 (46.3%)	34 (47.9%)
**Response rate in RAS unknown (N=14)**		
Responder (CR+PR) No. (%)	1 (14.3%)	2 (28.6%)
Not Responder (SD+PD) No. (%)	6 (85.7%)	5 (71.4%)

© 2021 Avallone A et al. *JAMA Network Open*.

eTable 3. Metastases-Resected Patients

	Standard Arm (n 115)	Experimental Arm (n 114)
**Patients having post-treatment surgery, n (%)**	42 (36.5%)	39 (34.2%)
**Pathological classification, n**		
R0	32	26
R1	5	2
R2	-	2
Unknown	5	9

© 2021 Avallone A et al. *JAMA Network Open*.

eTable 4. Postprogression Therapies According to Treatment Arm

	**Standard (n 115)**	**Experimental (n 115)**	
**LINE of treatment**	n (%)	n (%)	***P*[a]**
**Second**	79 (68.7%)	80 (69.6%)	0.05
Anti-EGFR	31	21	
Anti-VEGF	31	49	
Other	17	10	
**Third**	37 (32.2%)	38 (33.3%)	0.34
Anti-EGFR	6	12	
Anti-VEGF	23	22	
Other	8	4	
**Fourth**	9 (7.8%)	9 (7.8%)	0.64
Anti-VEGF	5	3	
Other	4	6	

[a]Chi-square test

© 2021 Avallone A et al. *JAMA Network Open*.

eTable 5. Worst per Patient Adverse Events Reported During Treatment, Classified According to the CTCAE, Version 4.0

	Standard												Experimental											
**CTCAE Term**	**G 0**	**%**	**G 1**	**%**	**G 2**	**%**	**G 3**	**%**	**G 4**	**%**	**G 5**	**%**	**G 0**	**%**	**G 1**	**%**	**G 2**	**%**	**G 3**	**%**	**G 4**	**%**	**G 5**	**%**
**Any Adverse Event**	2	(1,7)	11	(9,6)	27	(23,5)	58	(50,4)	15	(13,0)	2	(1,7)	5	(4,4)	14	(12,4)	29	(25,7)	51	(45,1)	12	(10,6)	2	(1,8)
**Blood and lymphatic system disorders**																								
Any adverse event in the category	88	(76,5)	17	(14,8)	7	(6,1)	1	(0,9)	2	(1,7)	0	(0,0)	84	(74,3)	16	(14,2)	9	(8,0)	4	(3,5)	0	(0,0)	0	(0,0)
Anemia	88	(76,5)	18	(15,7)	7	(6,1)	2	(1,7)	0	(0,0)	0	(0,0)	85	(75,2)	16	(14,2)	9	(8,0)	3	(2,7)	0	(0,0)	0	(0,0)
Febrile neutropenia	113	(98,3)	0	(0,0)	0	(0,0)	0	(0,0)	2	(1,7)	0	(0,0)	112	(99,1)	0	(0,0)	0	(0,0)	1	(0,9)	0	(0,0)	0	(0,0)
**Cardiac disorders**																								
Any adverse event in the category	115	(100,0)	0	(0,0)	0	(0,0)	0	(0,0)	0	(0,0)	0	(0,0)	109	(96,5)	1	(0,9)	2	(1,8)	1	(0,9)	0	(0,0)	0	(0,0)
Acute coronary syndrome	115	(100,0)	0	(0,0)	0	(0,0)	0	(0,0)	0	(0,0)	0	(0,0)	112	(99,1)	0	(0,0)	1	(0,9)	0	(0,0)	0	(0,0)	0	(0,0)
Atrial fibrillation	115	(100,0)	0	(0,0)	0	(0,0)	0	(0,0)	0	(0,0)	0	(0,0)	112	(99,1)	0	(0,0)	0	(0,0)	1	(0,9)	0	(0,0)	0	(0,0)
Palpitations	115	(100,0)	0	(0,0)	0	(0,0)	0	(0,0)	0	(0,0)	0	(0,0)	112	(99,1)	1	(0,9)	0	(0,0)	0	(0,0)	0	(0,0)	0	(0,0)
Other	115	(100,0)	0	(0,0)	0	(0,0)	0	(0,0)	0	(0,0)	0	(0,0)	112	(99,1)	0	(0,0)	1	(0,9)	0	(0,0)	0	(0,0)	0	(0,0)
**Ear and labyrinth disorders**																								
Any adverse event in the category	115	(100,0)	0	(0,0)	0	(0,0)	0	(0,0)	0	(0,0)	0	(0,0)	112	(99,1)	1	(0,9)	0	(0,0)	0	(0,0)	0	(0,0)	0	(0,0)
Hearing impaired	115	(100,0)	0	(0,0)	0	(0,0)	0	(0,0)	0	(0,0)	0	(0,0)	112	(99,1)	1	(0,9)	0	(0,0)	0	(0,0)	0	(0,0)	0	(0,0)
**Eye disorders**																								
Any adverse event in the category	115	(100,0)	0	(0,0)	0	(0,0)	0	(0,0)	0	(0,0)	0	(0,0)	112	(99,1)	0	(0,0)	0	(0,0)	1	(0,9)	0	(0,0)	0	(0,0)
Retinal vascular disorder	115	(100,0)	0	(0,0)	0	(0,0)	0	(0,0)	0	(0,0)	0	(0,0)	112	(99,1)	0	(0,0)	0	(0,0)	1	(0,9)	0	(0,0)	0	(0,0)
**Gastrointestinal disorders**																								
Any adverse event in the category	37	(32,2)	25	(21,7	24	(20,9	24	(20,9	4	(3,5)	1	(0,9	43	(38,1)	29	(25,7	22	(19,5	13	(11,5	4	(3,5)	2	(1,8

© 2021 Avallone A et al. *JAMA Network Open*.

	Standard													Experimental											
CTCAE Term	G0	%	G1	%	G2	%	G3	%	G4	%	G5	%		G0	%	G1	%	G2	%	G3	%	G4	%	G5	%
				)		)		)				)					)		)		)				)
Abdominal pain	105	(91,3)	4	(3,5)	1	(0,9)	5	(4,3)	0	(0,0)	0	(0,0)		110	(97,3)	1	(0,9)	0	(0,0)	2	(1,8)	0	(0,0)	0	(0,0)
Anal fistula	114	(99,1)	0	(0,0)	0	(0,0)	1	(0,9)	0	(0,0)	0	(0,0)		114	(100,0)	0	(0,0)	0	(0,0)	0	(0,0)	0	(0,0)	0	(0,0)
Ascites	114	(99,1)	0	(0,0)	1	(0,9)	0	(0,0)	0	(0,0)	0	(0,0)		114	(100,0)	0	(0,0)	0	(0,0)	0	(0,0)	0	(0,0)	0	(0,0)
Colitis	112	(97,4)	1	(0,9)	2	(1,7)	0	(0,0)	0	(0,0)	0	(0,0)		112	(99,1)	1	(0,9)	0	(0,0)	0	(0,0)	0	(0,0)	0	(0,0)
Colonic fistula	114	(99,1)	0	(0,0)	0	(0,0)	0	(0,0)	1	(0,9)	0	(0,0)		114	(100,0)	0	(0,0)	0	(0,0)	0	(0,0)	0	(0,0)	0	(0,0)
Colonic hemorrhage	114	(99,1)	0	(0,0)	1	(0,9)	0	(0,0)	0	(0,0)	0	(0,0)		114	(100,0)	0	(0,0)	0	(0,0)	0	(0,0)	0	(0,0)	0	(0,0)
Colonic obstruction	114	(99,1)	0	(0,0)	0	(0,0)	1	(0,9)	0	(0,0)	0	(0,0)		112	(99,1)	0	(0,0)	0	(0,0)	0	(0,0)	1	(0,9)	0	(0,0)
Colonic perforation	115	(100,0)	0	(0,0)	0	(0,0)	0	(0,0)	0	(0,0)	0	(0,0)		112	(99,1)	0	(0,0)	0	(0,0)	0	(0,0)	0	(0,0)	1	(0,9)
Constipation	111	(96,5)	2	(1,7)	1	(0,9)	1	(0,9)	0	(0,0)	0	(0,0)		110	(97,3)	2	(1,8)	1	(0,9)	0	(0,0)	0	(0,0)	0	(0,0)
Dental caries	115	(100,0)	0	(0,0)	0	(0,0)	0	(0,0)	0	(0,0)	0	(0,0)		112	(99,1)	0	(0,0)	0	(0,0)	1	(0,9)	0	(0,0)	0	(0,0)
Diarrhea	71	(61,7)	18	(15,7)	7	(6,1)	16	(13,9)	3	(2,6)	0	(0,0)		86	(76,1)	12	(10,6)	9	(8,0)	4	(3,5)	2	(1,8)	0	(0,0)
Esophageal pain	114	(99,1)	1	(0,9)	0	(0,0)	0	(0,0)	0	(0,0)	0	(0,0)		114	(100,0)	0	(0,0)	0	(0,0)	0	(0,0)	0	(0,0)	0	(0,0)
Flatulence	115	(100,0)	0	(0,0)	0	(0,0)	0	(0,0)	0	(0,0)	0	(0,0)		112	(99,1)	0	(0,0)	1	(0,9)	0	(0,0)	0	(0,0)	0	(0,0)
Ileal perforation	114	(99,1)	0	(0,0)	0	(0,0)	0	(0,0)	0	(0,0)	1	(0,9)		114	(100,0)	0	(0,0)	0	(0,0)	0	(0,0)	0	(0,0)	0	(0,0)
Mucositis oral	81	(70,4)	17	(14,8)	11	(9,6)	6	(5,2)	0	(0,0)	0	(0,0)		87	(77,0)	15	(13,3)	7	(6,2)	4	(3,5)	0	(0,0)	0	(0,0)
Nausea	71	(61,7)	22	(19,1)	14	(12,2)	8	(7,0)	0	(0,0)	0	(0,0)		69	(61,1)	29	(25,7)	13	(11,5)	2	(1,8)	0	(0,0)	0	(0,0)
Rectal fistula	114	(99,1)	0	(0,0)	0	(0,0)	1	(0,9)	0	(0,0)	0	(0,0)		114	(100,0)	0	(0,0)	0	(0,0)	0	(0,0)	0	(0,0)	0	(0,0)
Rectal hemorrhage	11	(100,	0	(0,0)	0	(0,0)	0	(0,0)	0	(0,0)	0	(0,0		11	(99,1)	1	(0,9)	0	(0,0)	0	(0,0)	0	(0,0)	0	(0,0

© 2021 Avallone A et al. *JAMA Network Open*.

	Standard													Experimental											
**CTCAE Term**	**G0**	**%**	**G1**	**%**	**G2**	**%**	**G3**	**%**	**G4**	**%**	**G5**	**%**		**G0**	**%**	**G1**	**%**	**G2**	**%**	**G3**	**%**	**G4**	**%**	**G5**	**%**
	5	0)										)		2											)
Rectal perforation	115	(100,0)	0	(0,0)	0	(0,0)	0	(0,0)	0	(0,0)	0	(0,0)		112	(99,1)	0	(0,0)	0	(0,0)	0	(0,0)	0	(0,0)	1	(0,9)
Salivary duct inflammation	114	(99,1)	1	(0,9)	0	(0,0)	0	(0,0)	0	(0,0)	0	(0,0)		114	(100,0)	0	(0,0)	0	(0,0)	0	(0,0)	0	(0,0)	0	(0,0)
Tooth discoloration	115	(100,0)	0	(0,0)	0	(0,0)	0	(0,0)	0	(0,0)	0	(0,0)		112	(99,1)	1	(0,9)	0	(0,0)	0	(0,0)	0	(0,0)	0	(0,0)
Vomiting	105	(91,3)	5	(4,3)	4	(3,5)	1	(0,9)	0	(0,0)	0	(0,0)		107	(94,7)	4	(3,5)	1	(0,9)	1	(0,9)	0	(0,0)	0	(0,0)
Other	115	(100,0)	0	(0,0)	0	(0,0)	0	(0,0)	0	(0,0)	0	(0,0)		112	(99,1)	0	(0,0)	0	(0,0)	0	(0,0)	1	(0,9)	0	(0,0)
**General disorders**																									
Any adverse event in the category	74	(64,3)	11	(9,6)	20	(17,4)	9	(7,8)	0	(0,0)	1	(0,9)		84	(74,3)	15	(13,3)	9	(8,0)	5	(4,4)	0	(0,0)	0	(0,0)
Death NOS	114	(99,1)	0	(0,0)	0	(0,0)	0	(0,0)	0	(0,0)	1	(0,9)		114	(100,0)	0	(0,0)	0	(0,0)	0	(0,0)	0	(0,0)	0	(0,0)
Edema limbs	115	(100,0)	0	(0,0)	0	(0,0)	0	(0,0)	0	(0,0)	0	(0,0)		112	(99,1)	1	(0,9)	0	(0,0)	0	(0,0)	0	(0,0)	0	(0,0)
Fatigue	85	(73,9)	10	(8,7)	15	(13,0)	5	(4,3)	0	(0,0)	0	(0,0)		97	(85,8)	9	(8,0)	3	(2,7)	4	(3,5)	0	(0,0)	0	(0,0)
Fever	108	(93,9)	1	(0,9)	5	(4,3)	1	(0,9)	0	(0,0)	0	(0,0)		105	(92,9)	2	(1,8)	5	(4,4)	1	(0,9)	0	(0,0)	0	(0,0)
Pain	112	(97,4)	0	(0,0)	2	(1,7)	1	(0,9)	0	(0,0)	0	(0,0)		108	(95,6)	3	(2,7)	1	(0,9)	1	(0,9)	0	(0,0)	0	(0,0)
Other	108	(93,9)	5	(4,3)	0	(0,0)	2	(1,7)	0	(0,0)	0	(0,0)		111	(98,2)	1	(0,9)	1	(0,9)	0	(0,0)	0	(0,0)	0	(0,0)
**Hepatobiliary disorders**																									
Any adverse event in the category	114	(99,1)	0	(0,0)	0	(0,0)	1	(0,9)	0	(0,0)	0	(0,0)		114	(100,0)	0	(0,0)	0	(0,0)	0	(0,0)	0	(0,0)	0	(0,0)
Other	114	(99,1)	0	(0,0)	0	(0,0)	1	(0,9)	0	(0,0)	0	(0,0)		114	(100,0)	0	(0,0)	0	(0,0)	0	(0,0)	0	(0,0)	0	(0,0)
**Immune system disorders**																									
Any adverse event in the category	111	(96,5)	0	(0,0)	0	(0,0)	3	(2,6)	1	(0,9)	0	(0,0)		110	(97,3)	0	(0,0)	1	(0,9)	1	(0,9)	1	(0,9)	0	(0,0)

© 2021 Avallone A et al. *JAMA Network Open*.

CTCAE Term	G0	%	G1	%	G2	%	G3	%	G4	%	G5	%		G0	%	G1	%	G2	%	G3	%	G4	%	G5	%
	**Standard**													**Experimental**											
Allergic reaction	111	(96,5)	0	(0,0)	0	(0,0)	3	(2,6)	1	(0,9)	0	(0,0)		110	(97,3)	0	(0,0)	1	(0,9)	1	(0,9)	1	(0,9)	0	(0,0)
**Infections and infestations**																									
Any adverse event in the category	112	(97,4)	0	(0,0)	1	(0,9)	2	(1,7)	0	(0,0)	0	(0,0)		110	(97,3)	1	(0,9)	1	(0,9)	1	(0,9)	0	(0,0)	0	(0,0)
Bronchial infection	115	(100,0)	0	(0,0)	0	(0,0)	0	(0,0)	0	(0,0)	0	(0,0)		112	(99,1)	0	(0,0)	1	(0,9)	0	(0,0)	0	(0,0)	0	(0,0)
Abdominal infection	114	(99,1)	0	(0,0)	0	(0,0)	1	(0,9)	0	(0,0)	0	(0,0)		114	(100,0)	0	(0,0)	0	(0,0)	0	(0,0)	0	(0,0)	0	(0,0)
Pelvic infection	115	(100,0)	0	(0,0)	0	(0,0)	0	(0,0)	0	(0,0)	0	(0,0)		112	(99,1)	0	(0,0)	0	(0,0)	1	(0,9)	0	(0,0)	0	(0,0)
Skin infection	114	(99,1)	0	(0,0)	0	(0,0)	1	(0,9)	0	(0,0)	0	(0,0)		114	(100,0)	0	(0,0)	0	(0,0)	0	(0,0)	0	(0,0)	0	(0,0)
Other	114	(99,1)	0	(0,0)	1	(0,9)	0	(0,0)	0	(0,0)	0	(0,0)		112	(99,1)	1	(0,9)	0	(0,0)	0	(0,0)	0	(0,0)	0	(0,0)
**Investigations**																									
Any adverse event in the category	41	(35,7)	21	(18,3)	19	(16,5)	27	(23,5)	7	(6,1)	0	(0,0)		44	(38,9)	28	(24,8)	13	(11,5)	21	(18,6)	7	(6,2)	0	(0,0)
ALT increased	92	(80,0)	19	(16,5)	3	(2,6)	1	(0,9)	0	(0,0)	0	(0,0)		90	(79,6)	18	(15,9)	4	(3,5)	1	(0,9)	0	(0,0)	0	(0,0)
ALP increased	92	(80,0)	19	(16,5)	3	(2,6)	1	(0,9)	0	(0,0)	0	(0,0)		91	(80,5)	17	(15,0)	4	(3,5)	1	(0,9)	0	(0,0)	0	(0,0)
ALT increased	115	(100,0)	0	(0,0)	0	(0,0)	0	(0,0)	0	(0,0)	0	(0,0)		111	(98,2)	2	(1,8)	0	(0,0)	0	(0,0)	0	(0,0)	0	(0,0)
Bilirubin increased	112	(97,4)	0	(0,0)	0	(0,0)	1	(0,9)	2	(1,7)	0	(0,0)		112	(99,1)	0	(0,0)	0	(0,0)	1	(0,9)	0	(0,0)	0	(0,0)
Neutrophil count decreased	71	(61,7)	5	(4,3)	11	(9,6)	23	(20,0)	5	(4,3)	0	(0,0)		71	(62,8)	12	(10,6)	7	(6,2)	16	(14,2)	7	(6,2)	0	(0,0)
Platelet count decreased	83	(72,2)	20	(17,4)	11	(9,6)	1	(0,9)	0	(0,0)	0	(0,0)		93	(82,3)	10	(8,8)	8	(7,1)	2	(1,8)	0	(0,0)	0	(0,0)
White blood cell decreased	80	(69,6)	17	(14,8)	10	(8,7)	7	(6,1)	1	(0,9)	0	(0,0)		85	(75,2)	12	(10,6)	11	(9,7)	3	(2,7)	2	(1,8)	0	(0,0)
Other	115	(100,0)	0	(0,0)	0	(0,0)	0	(0,0)	0	(0,0)	0	(0,0)		112	(99,1)	1	(0,9)	0	(0,0)	0	(0,0)	0	(0,0)	0	(0,0)
**Metabolism and nutrition disorders**																									

© 2021 Avallone A et al. *JAMA Network Open*.

	Standard													Experimental											
**CTCAE Term**	**G0**	**%**	**G1**	**%**	**G2**	**%**	**G3**	**%**	**G4**	**%**	**G5**	**%**		**G0**	**%**	**G1**	**%**	**G2**	**%**	**G3**	**%**	**G4**	**%**	**G5**	**%**
Any adverse event in the category	113	(98,3)	1	(0,9)	0	(0,0)	1	(0,9)	0	(0,0)	0	(0,0)		111	(98,2)	0	(0,0)	1	(0,9)	0	(0,0)	1	(0,9)	0	(0,0)
Dehydration	115	(100,0)	0	(0,0)	0	(0,0)	0	(0,0)	0	(0,0)	0	(0,0)		112	(99,1)	0	(0,0)	1	(0,9)	0	(0,0)	0	(0,0)	0	(0,0)
Hypertriglyceridemia	114	(99,1)	0	(0,0)	0	(0,0)	1	(0,9)	0	(0,0)	0	(0,0)		112	(99,1)	0	(0,0)	0	(0,0)	0	(0,0)	1	(0,9)	0	(0,0)
Other	114	(99,1)	1	(0,9)	0	(0,0)	0	(0,0)	0	(0,0)	0	(0,0)		114	(100,0)	0	(0,0)	0	(0,0)	0	(0,0)	0	(0,0)	0	(0,0)
**Nervous system disorders**																									
Any adverse event in the category	45	(39,1)	26	(22,6)	28	(24,3)	15	(13,0)	1	(0,9)	0	(0,0)		44	(38,9)	27	(23,9)	23	(20,4)	19	(16,8)	0	(0,0)	0	(0,0)
Cognitive disturbance	115	(100,0)	0	(0,0)	0	(0,0)	0	(0,0)	0	(0,0)	0	(0,0)		112	(99,1)	0	(0,0)	0	(0,0)	1	(0,9)	0	(0,0)	0	(0,0)
Dizziness	115	(100,0)	0	(0,0)	0	(0,0)	0	(0,0)	0	(0,0)	0	(0,0)		111	(98,2)	0	(0,0)	1	(0,9)	1	(0,9)	0	(0,0)	0	(0,0)
Dysesthesia	106	(92,2)	4	(3,5)	4	(3,5)	1	(0,9)	0	(0,0)	0	(0,0)		109	(96,5)	1	(0,9)	1	(0,9)	2	(1,8)	0	(0,0)	0	(0,0)
Dysgeusia	114	(99,1)	1	(0,9)	0	(0,0)	0	(0,0)	0	(0,0)	0	(0,0)		110	(97,3)	3	(2,7)	0	(0,0)	0	(0,0)	0	(0,0)	0	(0,0)
Neuralgia	114	(99,1)	0	(0,0)	0	(0,0)	1	(0,9)	0	(0,0)	0	(0,0)		111	(98,2)	1	(0,9)	0	(0,0)	1	(0,9)	0	(0,0)	0	(0,0)
Paresthesia	86	(74,8)	11	(9,6)	8	(7,0)	10	(8,7)	0	(0,0)	0	(0,0)		87	(77,0)	12	(10,6)	6	(5,3)	8	(7,1)	0	(0,0)	0	(0,0)
Peripheral motor neuropathy	112	(97,4)	2	(1,7)	1	(0,9)	0	(0,0)	0	(0,0)	0	(0,0)		111	(98,2)	1	(0,9)	0	(0,0)	1	(0,9)	0	(0,0)	0	(0,0)
Peripheral sensory neuropathy	67	(58,3)	19	(16,5)	24	(20,9)	5	(4,3)	0	(0,0)	0	(0,0)		69	(61,1)	19	(16,8)	20	(17,7)	5	(4,4)	0	(0,0)	0	(0,0)
Stroke	114	(99,1)	0	(0,0)	0	(0,0)	0	(0,0)	1	(0,9)	0	(0,0)		112	(99,1)	0	(0,0)	1	(0,9)	0	(0,0)	0	(0,0)	0	(0,0)
Syncope	115	(100,0)	0	(0,0)	0	(0,0)	0	(0,0)	0	(0,0)	0	(0,0)		111	(98,2)	0	(0,0)	0	(0,0)	2	(1,8)	0	(0,0)	0	(0,0)
Transient ischemic attacks	114	(99,1)	1	(0,9)	0	(0,0)	0	(0,0)	0	(0,0)	0	(0,0)		114	(100,0)	0	(0,0)	0	(0,0)	0	(0,0)	0	(0,0)	0	(0,0)
Other	115	(100,0)	0	(0,0)	0	(0,0)	0	(0,0)	0	(0,0)	0	(0,0)		112	(99,1)	1	(0,9)	0	(0,0)	0	(0,0)	0	(0,0)	0	(0,0)

© 2021 Avallone A et al. *JAMA Network Open*.

	Standard												Experimental											
**CTCAE Term**	**G0**	**%**	**G1**	**%**	**G2**	**%**	**G3**	**%**	**G4**	**%**	**G5**	**%**	**G0**	**%**	**G1**	**%**	**G2**	**%**	**G3**	**%**	**G4**	**%**	**G5**	**%**
**Psychiatric disorders**																								
Any adverse event in the category	114	(99,1)	0	(0,0)	1	(0,9)	0	(0,0)	0	(0,0)	0	(0,0)	114	(100,0)	0	(0,0)	0	(0,0)	0	(0,0)	0	(0,0)	0	(0,0)
Anxiety	114	(99,1)	0	(0,0)	1	(0,9)	0	(0,0)	0	(0,0)	0	(0,0)	114	(100,0)	0	(0,0)	0	(0,0)	0	(0,0)	0	(0,0)	0	(0,0)
**Renal and urinary disorders**																								
Any adverse event in the category	111	(96,5)	3	(2,6)	1	(0,9)	0	(0,0)	0	(0,0)	0	(0,0)	100	(88,5)	13	(11,5)	0	(0,0)	0	(0,0)	0	(0,0)	0	(0,0)
Hematuria	115	(100,0)	0	(0,0)	0	(0,0)	0	(0,0)	0	(0,0)	0	(0,0)	110	(97,3)	3	(2,7)	0	(0,0)	0	(0,0)	0	(0,0)	0	(0,0)
Proteinuria	112	(97,4)	2	(1,7)	1	(0,9)	0	(0,0)	0	(0,0)	0	(0,0)	103	(91,2)	10	(8,8)	0	(0,0)	0	(0,0)	0	(0,0)	0	(0,0)
Other	114	(99,1)	1	(0,9)	0	(0,0)	0	(0,0)	0	(0,0)	0	(0,0)	111	(98,2)	2	(1,8)	0	(0,0)	0	(0,0)	0	(0,0)	0	(0,0)
**Respiratory, thoracic and mediastinal disorders**																								
Any adverse event in the category	99	(86,1)	13	(11,3)	3	(2,6)	0	(0,0)	0	(0,0)	0	(0,0)	90	(79,6)	18	(15,9)	3	(2,7)	2	(1,8)	0	(0,0)	0	(0,0)
Epistaxis	108	(93,9)	7	(6,1)	0	(0,0)	0	(0,0)	0	(0,0)	0	(0,0)	98	(86,7)	14	(12,4)	1	(0,9)	0	(0,0)	0	(0,0)	0	(0,0)
Hiccups	113	(98,3)	1	(0,9)	1	(0,9)	0	(0,0)	0	(0,0)	0	(0,0)	110	(97,3)	3	(2,7)	0	(0,0)	0	(0,0)	0	(0,0)	0	(0,0)
Hoarseness	115	(100,0)	0	(0,0)	0	(0,0)	0	(0,0)	0	(0,0)	0	(0,0)	112	(99,1)	1	(0,9)	0	(0,0)	0	(0,0)	0	(0,0)	0	(0,0)
Laryngospasm	115	(100,0)	0	(0,0)	0	(0,0)	0	(0,0)	0	(0,0)	0	(0,0)	112	(99,1)	0	(0,0)	0	(0,0)	1	(0,9)	0	(0,0)	0	(0,0)
Voice alteration	109	(94,8)	5	(4,3)	1	(0,9)	0	(0,0)	0	(0,0)	0	(0,0)	106	(93,8)	5	(4,4)	2	(1,8)	0	(0,0)	0	(0,0)	0	(0,0)
Other	113	(98,3)	1	(0,9)	1	(0,9)	0	(0,0)	0	(0,0)	0	(0,0)	112	(99,1)	0	(0,0)	0	(0,0)	1	(0,9)	0	(0,0)	0	(0,0)
**Skin and subcutaneous tissue disorders**																								
Any adverse event in the category	86	(74,8)	12	(10,4)	13	(11,3)	4	(3,5)	0	(0,0)	0	(0,0)	94	(83,2)	4	(3,5)	10	(8,8)	5	(4,4)	0	(0,0)	0	(0,0)
Alopecia	113	(98,3)	1	(0,9)	1	(0,9)	0	(0,0)	0	(0,0)	0	(0,0)	114	(100,0)	0	(0,0)	0	(0,0)	0	(0,0)	0	(0,0)	0	(0,0)

© 2021 Avallone A et al. *JAMA Network Open*.

CTCAE Term	Standard G0	%	G1	%	G2	%	G3	%	G4	%	G5	%		Experimental G0	%	G1	%	G2	%	G3	%	G4	%	G5	%
Bullous dermatitis	114	(99,1)	0	(0,0)	1	(0,9)	0	(0,0)	0	(0,0)	0	(0,0)		114	(100,0)	0	(0,0)	0	(0,0)	0	(0,0)	0	(0,0)	0	(0,0)
Palmar-plantar erythrodysesthesia syndrome	92	(80,0)	9	(7,8)	10	(8,7)	4	(3,5)	0	(0,0)	0	(0,0)		94	(83,2)	4	(3,5)	10	(8,8)	5	(4,4)	0	(0,0)	0	(0,0)
Pruritus	112	(97,4)	3	(2,6)	0	(0,0)	0	(0,0)	0	(0,0)	0	(0,0)		114	(100,0)	0	(0,0)	0	(0,0)	0	(0,0)	0	(0,0)	0	(0,0)
Skin ulceration	114	(99,1)	0	(0,0)	1	(0,9)	0	(0,0)	0	(0,0)	0	(0,0)		114	(100,0)	0	(0,0)	0	(0,0)	0	(0,0)	0	(0,0)	0	(0,0)
**Vascular disorders**																									
Any adverse event in the category	84	(73,0)	8	(7,0)	13	(11,3)	9	(7,8)	1	(0,9)	0	(0,0)		78	(69,0)	13	(11,5)	16	(14,2)	6	(5,3)	0	(0,0)	0	(0,0)
Hypertension	90	(78,3)	7	(6,1)	11	(9,6)	7	(6,1)	0	(0,0)	0	(0,0)		81	(71,7)	13	(11,5)	16	(14,2)	3	(2,7)	0	(0,0)	0	(0,0)
Hypotension	115	(100,0)	0	(0,0)	0	(0,0)	0	(0,0)	0	(0,0)	0	(0,0)		112	(99,1)	0	(0,0)	0	(0,0)	1	(0,9)	0	(0,0)	0	(0,0)
Phlebitis	114	(99,1)	0	(0,0)	1	(0,9)	0	(0,0)	0	(0,0)	0	(0,0)		114	(100,0)	0	(0,0)	0	(0,0)	0	(0,0)	0	(0,0)	0	(0,0)
Superficial thrombophlebitis	114	(99,1)	0	(0,0)	1	(0,9)	0	(0,0)	0	(0,0)	0	(0,0)		114	(100,0)	0	(0,0)	0	(0,0)	0	(0,0)	0	(0,0)	0	(0,0)
Thromboembolic event	109	(94,8)	1	(0,9)	2	(1,7)	2	(1,7)	1	(0,9)	0	(0,0)		111	(98,2)	0	(0,0)	0	(0,0)	2	(1,8)	0	(0,0)	0	(0,0)
Other	114	(99,1)	1	(0,9)	0	(0,0)	0	(0,0)	0	(0,0)	0	(0,0)		110	(97,3)	2	(1,8)	1	(0,9)	0	(0,0)	0	(0,0)	0	(0,0)

eTable 6. Distribution of Best QOL Response From Baseline With EORTC QLQ-C30 Global Health Status, Functional Scales, and Symptom Items

	Standard							Experimental							
Domain/Item	Improved		Stable		Worse			Improved		Stable		Worse			***P***[a]
Global health status	33	(42,9%)	20	(26,0%)	24	(31,2%)		40	(46,5%)	27	(31,4%)	19	(22,1%)		0,41
Physical functioning	14	(18,2%)	32	(41,6%)	31	(40,3%)		26	(30,2%)	40	(46,5%)	20	(23,3%)		0,04

© 2021 Avallone A et al. *JAMA Network Open*.

Role functioning	21	(27,3%)	30	(39,0%)	26	(33,8%)		27	(31,8%)	32	(37,6%)	26	(30,6%)		0,81
Emotional functioning	34	(44,2%)	30	(39,0%)	13	(16,9%)		41	(47,7%)	34	(39,5%)	11	(12,8%)		0,75
Cognitive functioning	23	(29,9%)	43	(55,8%)	11	(14,3%)		21	(24,4%)	53	(61,6%)	12	(14,0%)		0,71
Social functioning	31	(40,3%)	33	(42,9%)	13	(16,9%)		29	(33,7%)	49	(57,0%)	8	(9,3%)		0,14
Fatigue	27	(35,1%)	14	(18,2%)	36	(46,8%)		33	(38,4%)	18	(20,9%)	35	(40,7%)		0,73
Nausea/vomiting	16	(20,8%)	35	(45,5%)	26	(33,8%)		17	(19,8%)	39	(45,3%)	30	(34,9%)		0,98
Pain	32	(41,6%)	23	(29,9%)	22	(28,6%)		43	(50,0%)	26	(30,2%)	17	(19,8%)		0,38
Dyspnoea	18	(23,4%)	50	(64,9%)	9	(11,7%)		18	(20,9%)	60	(69,8%)	8	(9,3%)		0,79
Insomnia	27	(35,1%)	36	(46,8%)	14	(18,2%)		37	(43,0%)	37	(43,0%)	12	(14,0%)		0,54
Appetite loss	20	(26,0%)	38	(49,4%)	19	(24,7%)		25	(29,1%)	42	(48,8%)	19	(22,1%)		0,88
Constipation	19	(24,7%)	45	(58,4%)	13	(16,9%)		34	(39,5%)	46	(53,5%)	6	(7,0%)		0,04
Diarrhoea	13	(16,9%)	49	(63,6%)	15	(19,5%)		17	(19,8%)	51	(59,3%)	18	(20,9%)		0,84
Financial difficulties	20	(26,0%)	48	(62,3%)	9	(11,7%)		27	(31,4%)	45	(52,3%)	14	(16,3%)		0,42

[a]Chi-square test

© 2021 Avallone A et al. *JAMA Network Open*.
