# Supplementary material for: Effect of Bevacizumab in Combination With Standard Oxaliplatin-Based Regimens in Patients With Metastatic Colorectal Cancer: A Randomized Clinical Trial
Source: JAMA Netw Open. 2021 Jul 26;4(7):e2118475. doi: 10.1001/jamanetworkopen.2021.18475 (PMC8314140; doi:10.1001/jamanetworkopen.2021.18475)
Supplement: Supplement 3. — Data Sharing Statement [file jamanetwopen-e2118475-s003.pdf]

Data Sharing Statement

Avallone. Effect of Bevacizumab in Combination With Standard Oxaliplatin-Based Regimens in Patients With Metastatic Colorectal Cancer. *JAMA Netw Open*. Published July 26, 2021. doi:10.1001/jamanetworkopen.2021.18475

Data
Data available: Yes
Data types: Deidentified participant data
How to access data: a.avallone@istitutotumori.na.it
When available: With publication

Supporting Documents
Document types: None

Additional Information
Who can access the data: researchers whose proposed use of the data has been approved
Types of analyses: for any purpose
Mechanisms of data availability: with investigator support and after approval of a proposal
